# Two modes of gene regulation by TFL1 mediate its dual function in flowering time and shoot determinacy of *Arabidopsis*

**DOI:** 10.1242/dev.202089

**Published:** 2023-12-07

**Authors:** Martina Cerise, Vítor da Silveira Falavigna, Gabriel Rodríguez-Maroto, Antoine Signol, Edouard Severing, He Gao, Annabel van Driel, Coral Vincent, Sandra Wilkens, Francesca Romana Iacobini, Pau Formosa-Jordan, Alice Pajoro, George Coupland

**Affiliations:** ^1^ Department of Plant Developmental Biology, Max Planck Institute for Plant Breeding Research, Cologne 50829, Germany; ^2^ Institute of Molecular Biology and Pathology, National Research Council, c/o Department Biology and Biotechnology ‘C. Darwin’ Sapienza University, Rome 00185, Italy

**Keywords:** Shoot meristem, Floral transition, Inflorescence development, FD, Transcriptomics, *Arabidopsis*

## Abstract

Plant organ primordia develop successively at the shoot apical meristem (SAM). In *Arabidopsis*, primordia formed early in development differentiate into vegetative leaves, whereas those formed later generate inflorescence branches and flowers. TERMINAL FLOWER 1 (TFL1), a negative regulator of transcription, acts in the SAM to delay flowering and to maintain inflorescence meristem indeterminacy. We used confocal microscopy, time-resolved transcript profiling and reverse genetics to elucidate this dual role of TFL1. We found that TFL1 accumulates dynamically in the SAM reflecting its dual function. Moreover, TFL1 represses two major sets of genes. One set includes genes that promote flowering, expression of which increases earlier in *tfl1* mutants. The other set is spatially misexpressed in *tfl1* inflorescence meristems. The misexpression of these two gene sets in *tfl1* mutants depends upon FD transcription factor, with which TFL1 interacts. Furthermore, the MADS-box gene *SEPALLATA 4*, which is upregulated in *tfl1*, contributes both to the floral transition and shoot determinacy defects of *tfl1* mutants. Thus, we delineate the dual function of TFL1 in shoot development in terms of its dynamic spatial distribution and different modes of gene repression.

## Teaser

**Highlighted Article:** TFL1, a transcriptional cofactor, regulates floral transition and shoot indeterminacy. This study quantifies the dynamic localisation of TFL1 and characterises its role in gene expression at these stages of reproductive development.

## INTRODUCTION

During plant shoot development, lateral organs are formed iteratively by the shoot apical meristem (SAM), which is located at the shoot tip. After germination, the SAM repeatedly gives rise to lateral vegetative structures such as leaves, whereas later it initiates the formation of flowers and inflorescences. The stage during shoot development at which the SAM transitions from vegetative to reproductive development is determined by environmental and endogenous signals that cause it to change identity from a vegetative to an inflorescence meristem (Andrés and Coupland, 2012; Kinoshita and Richter, 2020). In *Arabidopsis thaliana*, the inflorescence meristem is indeterminate and gives rise to a raceme, an inflorescence in which flowers are repeatedly formed on the axis of the stem (Alvarez et al., 1992; Bradley et al., 1997; Shannon and Meeks-Wagner, 1991). Before forming flowers, the *A. thaliana* inflorescence meristem forms a small number of leaves (cauline leaves) that also bear racemes in their axils. TERMINAL FLOWER 1 (TFL1) has a dual role in development of the *A. thaliana* raceme: it delays the transition to flowering, thereby increasing the number of vegetative leaves, and later it maintains indeterminacy of the inflorescence meristem. Therefore, mutations in *TFL1* both cause early flowering and convert the inflorescence meristem into a determinate flower, greatly reducing the number of flowers and branches formed in the inflorescence (Alvarez et al., 1992; Shannon and Meeks-Wagner, 1991). The role of TFL1 in repressing floral transition and how this differs from its function in maintaining inflorescence indeterminacy are not well understood.

*TFL1* encodes a small protein related to phosphatidylethanolamine-binding proteins that is expressed in the SAM (Bradley et al., 1997) and moves between meristematic cells (Conti and Bradley, 2007; Goretti et al., 2020). It forms a complex with the bZIP transcription factor FD that is also expressed in the meristem, and together they directly repress transcription of genes involved in floral development, such as *APETALA1* (*AP1*) (Abe et al., 2005; Collani et al., 2019; Gustafson-Brown et al., 1994; Hanano and Goto, 2011; Romera-Branchat et al., 2020; Wigge et al., 2005; Zhu et al., 2020). Recently, genome-wide analysis identified genomic loci with which TFL1 and FD are both associated (Goretti et al., 2020; Zhu et al., 2020). These sites include many genes involved in floral and inflorescence development, supporting the idea that FD and TFL1 coregulate a large set of genes in addition to *AP1*. Consistent with its role at this stage, *TFL1* transcription is increased in the inflorescence meristem by the MADS-box transcription factors SUPPRESSOR OF OVEREXPRESSION OF CONSTANS 1 (SOC1), AGAMOUS-LIKE 24 (AGL24) and XAANTAL 2 (XAL2) (Azpeitia et al., 2021; Perez-Ruiz et al., 2015; Serrano-Mislata et al., 2016). In contrast, in developing floral primordia, *TFL1* transcription is directly repressed by AP1 and its paralogue CAULIFLOWER (CAL), as well as other MADS-box transcription factors, including SOC1, AGL24, SHORT VEGETATIVE PHASE (SVP) and SEPALLATA 4 (SEP4), and this contributes to floral meristem identity (Bowman et al., 1993; Goslin et al., 2017; Kaufmann et al., 2010; Liljegren et al., 1999; Liu et al., 2013; Ratcliffe et al., 1999).

To repress floral transition in *Arabidopsis*, TFL1 is thought to antagonise the function of the paralogous proteins FLOWERING LOCUS T (FT) and TWIN SISTER OF FT (TSF). Although they are closely related to TFL1, FT and TSF promote floral transition (Kardailsky et al., 1999; Kobayashi et al., 1999; Yamaguchi et al., 2005). After being expressed in the vasculature, FT and TSF move to the SAM where they interact with FD (Abe et al., 2019; Corbesier et al., 2007; Jaeger and Wigge, 2007; Mathieu et al., 2007). Mutations in *FT* and *TSF* delay floral transition, and are epistatic to *tfl1* in the regulation of flowering time (Lee et al., 2019), demonstrating that FT and TSF are required for the early-flowering phenotype of *tfl1* mutants. Similar genetic interactions between orthologues of *TFL1* and *FT* have been described in the architecture of the tomato shoot and are important in the regulation of yield (Jiang et al., 2013; Shalit et al., 2009). The antagonism between TFL1 and FT/TSF may therefore be based on their competition for interaction with FD at the SAM, with TFL1 acting as a transcriptional corepressor whereas FT/TSF act as transcriptional coactivators (Ahn et al., 2006; Hanano and Goto, 2011; Jaeger et al., 2013; McGarry and Ayre, 2012). Here, we use a combination of confocal imaging, and genomic and transcriptomic approaches to analyse the dual role of TFL1 in regulating floral transition and in conferring inflorescence indeterminacy.

## RESULTS

### TFL1 acts during seedling development to repress the changes in meristem morphology that occur during floral transition

**Fig. 1.**
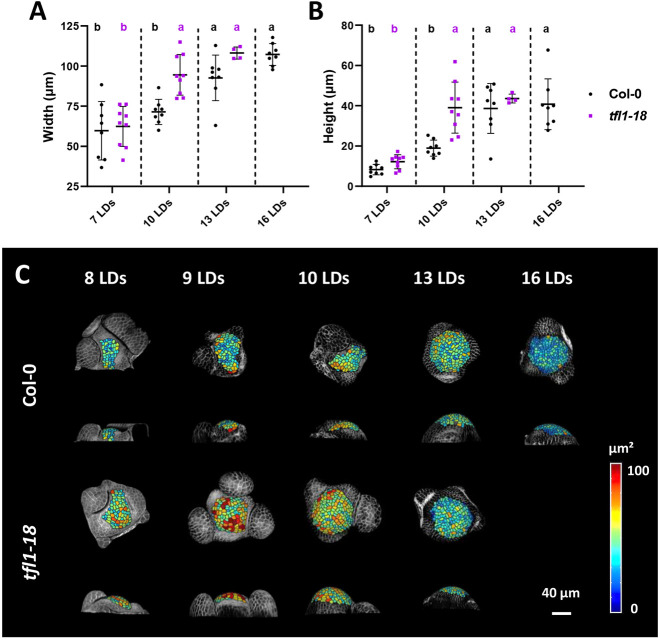
**Meristem size and shape in *tfl1-18* mutants and Col-0 during floral transition.** (A,B) Meristem shape measured by the width (A) and height (B) of Col-0 and *tfl1-18* meristems during floral transition. Statistically significant differences between means were analysed by one-way ANOVA followed by Tukey's multiple comparisons test. Lowercase letters are used to label means, such that bars bearing different letters are statistically different from one another with a minimum *P*-value of <0.05. Data are mean±s.d. (C) 2.5D segmentation of Col-0 and *tfl1-18* shoot apical meristems during floral transition performed with MorphoGraphX. Cells are colour coded according to their area. The *tfl1-18* mutant was not analysed at 16 LDs because by then the meristem was converted into a flower.

During floral transition, the SAM of *A. thaliana* changes in size and shape as it progresses from the vegetative to the inflorescence stage (Kinoshita et al., 2020). As part of this process, the SAM increases disproportionately in height with respect to width, creating a characteristic dome-like shaped meristem. To investigate the relationship between *TFL1* activity and SAM morphology, the height and width of Col-0 and *tfl1-18* SAMs were compared from the vegetative stage until floral primordia were formed (Fig. 1; Figs S1, S6; Materials and Methods). Doming of the SAM occurred at 10 days in long-day conditions (LDs; 16 h light/8 h dark) in *tfl1-18* and at 13 LDs in Col-0, and floral primordia appeared at 13 LDs and at 16 LDs, respectively (Fig. 1A,B; Fig. S6). To analyse meristem morphology at cellular resolution, the L1 cells of Col-0 and *tfl1-18* SAMs were segmented using MorphoGraphX software (Barbier de Reuille et al., 2015). At 8 LDs, meristem area and cell number did not differ between genotypes, but from 9 LDs onwards these parameters increased in *tfl1-18* and reached maxima at 10 LDs (Fig. 1C; Fig. S1). By contrast, in the SAM of Col-0, meristem area and cell number only started to increase at 13 LDs. These results indicate that at the beginning of floral transition, *TFL1* delays doming by ∼3 days and in *tfl1* mutants the changes in SAM morphology and the progression to floral development are accelerated.

### Stage-dependent variation in TFL1 distribution at the SAM correlates with its dual role in shoot development

**Fig. 2.**
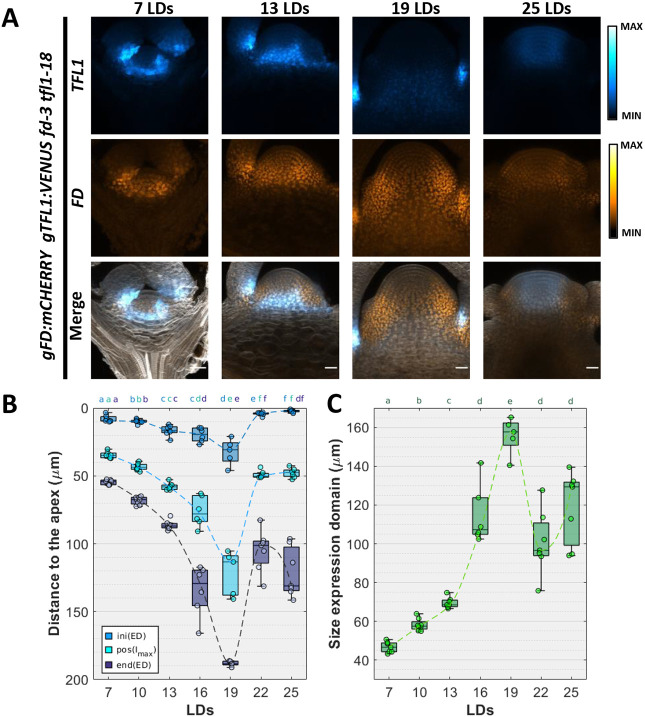
**Dynamic expression of TFL1 during floral transition.** (A) The expression of TFL1 (upper panels), FD (central panel) and merged (lower panel) in the *fd-3 tfl1-18* double mutant background grown under long days (LDs) from the vegetative stage (7 LDs) to the reproductive stage (25 LDs). Scale bars: 20 µm. (B) Box plot showing the distance of the TFL1 expression domain from the tip of the meristem at different developmental stages in LDs. Blue boxes indicate the apical boundary of the TFL1 expression domain [ini(ED)]; light-blue boxes indicate the maximum expression peak of the TFL1 domain [pos(I_max_)]; dark-blue boxes indicate the lower boundary of the TFL1 domain [end(ED)]. Mean values were compared using the Mann–Whitney U test. Lowercase letters are used to label means, such that bars bearing different letters are statistically different from one another with a minimum *P*-value of <0.05. (C) A box plot showing the area of the TFL1 domain at time points from the vegetative stage to the reproductive stage in LDs. Means were compared using the Mann–Whitney U test. Box plots indicate median values (middle bars) and first to third interquartile ranges (boxes).

*TFL1* mRNA is expressed weakly below the vegetative meristem and increased in abundance in a similar pattern below the dome of the inflorescence meristem (Bradley et al., 1997; Conti and Bradley, 2007) (Fig. S2). However, TFL1 protein can move between meristematic cells (Conti and Bradley, 2007; Goretti et al., 2020). Therefore, to analyse the temporal and spatial distribution of TFL1 throughout the floral transition, a *gTFL1:VENUS* marker line that complemented the phenotype of the *tfl1-18* mutant (Fig. S3) was constructed. TFL1-VENUS distribution in the SAM was then analysed during floral transition under LDs or short-day conditions (SDs) (Fig. 2; Figs S2, S4). Under both conditions, TFL1-VENUS was detected during the vegetative phase mainly below the meristem: at the doming stage its distribution appeared more diffuse, and in the inflorescence meristem it was present at the tip of the SAM (Fig. 2; Figs S2, S4). Therefore, irrespective of the photoperiod, the confocal imaging suggests two distinct patterns of accumulation during vegetative and inflorescence development, with a more diffuse pattern during floral transition. Because the function of TFL1 is proposed to depend on its interaction with FD (Goretti et al., 2020; Hanano and Goto, 2011; Zhu et al., 2020), colocalisation of TFL1-VENUS and mCHERRY-FD expressed from *mCHERRY:gFD* was analysed during floral transition. Comparison of mCHERRY-FD and TFL1-VENUS expression under LDs and SDs demonstrated that they overlapped below the meristem during vegetative development (Fig. 2A at 7 and 13 LDs; Fig. S2C at 2, 3, 4 weeks in SDs), whereas at doming (Fig. 2A at 19 LDs; Fig. S2C at 6 weeks in SDs), the deeper and more diffuse distribution of TFL1-VENUS reduced the extent of colocalisation of both proteins in the SAM (Fig. 2; Figs S2, S4). In the inflorescence meristem, TFL1 and FD expression overlapped again at the tip of the meristem in both LDs and SDs (Fig. 2; Figs S2, S4).

To examine the dynamic pattern of TFL1-VENUS expression in more detail, its fluorescence signals were quantified in the SAM from confocal images obtained under LDs (Supplementary Materials and Methods). The distribution of TFL1-VENUS was defined by the depth from the tip of the meristem to the position at which VENUS signal showed maximum intensity, and a threshold of 10% of the maximum fluorescence level was used to define the upper and lower limits of TFL1-VENUS expression. During the vegetative phase, when TFL1 delays the formation of the inflorescence meristem (7, 13 LDs in Fig. 2A), the peak in TFL1-VENUS fluorescence was present 40-60 µm from the tip and extended over a distance of ∼45-70 µm (7 LDs in Fig. 2B,C). At doming (19 LDs in Fig. 2A), the maximum fluorescence peak of TFL1-VENUS was present in deeper layers of the meristem ∼120 µm from the tip and in a more diffuse pattern extending over 160 µm (Fig. 2A-C; Fig. S4). During doming, strong TFL1-VENUS expression was detected in the axils of cauline leaves at the positions of axillary meristems, as previously observed (Conti and Bradley, 2007; Zhu et al., 2020), but this was excluded from the quantification (Supplementary Materials and Methods). At later time points, when the inflorescence meristem was established, TFL1-VENUS was detected broadly at the tip of the SAM and in the epidermis, with a peak of fluorescence at a depth of ∼50 µm and extending over approximately 130 µm (25 LDs in Fig. 2A-C; Fig. S4).

Taken together, these data demonstrate that TFL1 protein shows distinct distribution patterns during vegetative development, when it represses floral transition, and during inflorescence development, when it maintains indeterminacy of the SAM. Furthermore, the colocalisation of FD and TFL1 is dynamic so that they overlap in the vegetative and mature inflorescence meristems, but diverge during doming.

### Time-resolved transcriptomic analysis reveals two modes of flowering-gene regulation by TFL1

**Fig. 3.**
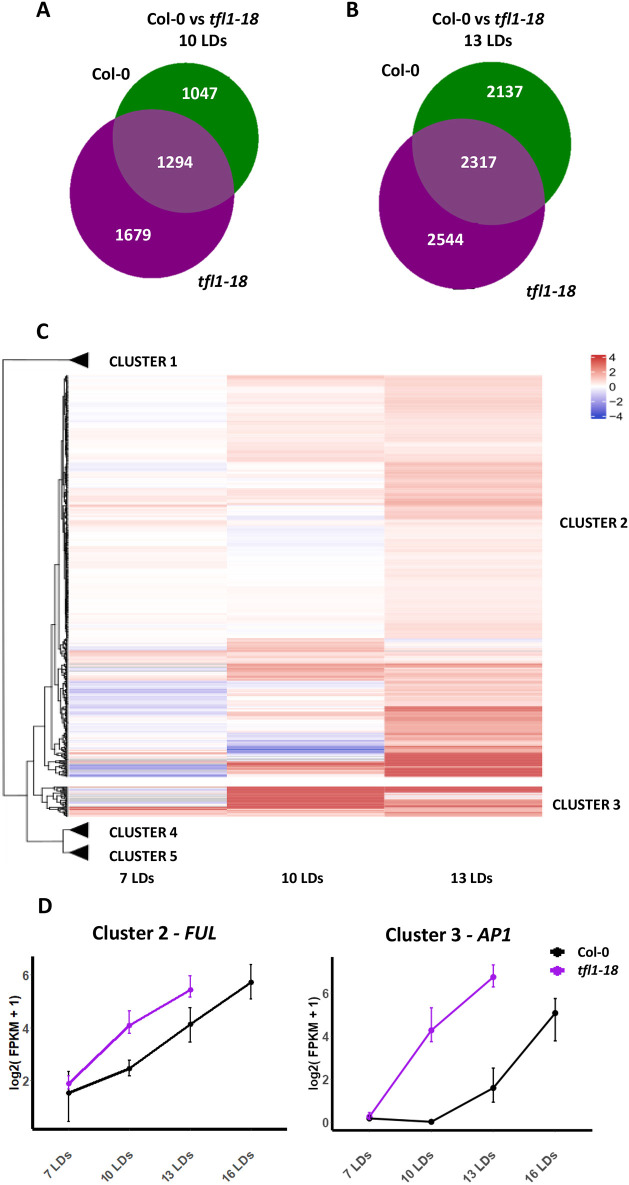
**Comparison of RNA-seq data from *tfl1-18* and Col-0 during floral transition.** (A,B) Venn diagrams showing the number of DEGs with respect to the vegetative stage (7 LDs) at 10 LDs (A) and 13 LDs (B) for Col-0 (green) and *tfl1-18* (purple). (C) Heat map of DEGs found only in *tfl1-18*. The comparison shows five identified clusters based on the fold change in expression between wild type and *tfl1-18* at 7, 10 and 13 LDs. Clusters 1, 4 and 5 are indicated by a triangle; the extended version is in Fig. S7. (D) Abundance of mRNAs of *FUL* and *AP1* in Col-0 and *tfl1-18* during floral transition based on the RNA-seq data. FPKM denotes fragments per kilobase of exon per million mapped fragments.

*TFL1* is expressed early in vegetative development and during floral transition. To understand its effect on gene regulation during these stages and the extent to which gene deregulation in *tfl1-18* mutants relies on FD activity, RNA-sequencing (RNA-seq) was performed on apical samples of Col-0, *tfl1-18*, *fd-3* and *tfl1-18 fd-3* every 3 days from the vegetative stage (7 LDs) until the establishment of the inflorescence meristem for each genotype [16 LDs (Col-0), 13 LDs (*tfl1-18*), 22 LDs (*fd-3*) and 22 LDs (*tfl1-18 fd-3*)]. At each time point, the developmental stage of each genotype was assessed by confocal microscopy (Fig. S6). Gene expression patterns in *tfl1-18* and Col-0 were first compared by principal component analysis and, at 7 LDs, there was no difference between the genotypes, consistent with the phenotypic analysis (Fig. S1; Tables S1 and S4; Fig. S6). Therefore, genes that change in expression between 7 LDs and each later time point were identified (Fig. S5; Fig. 3A,B). This analysis was carried out separately for each genotype and each time point (e.g. 7 LDs Col-0 versus 10 LDs Col-0; 7 LDs *tfl1-18* versus 10 LDs *tfl1-18*). The differentially expressed genes (DEGs) identified for Col-0 and *tfl1-18* at each time point were then compared. This comparison identified 1679 genes differentially expressed in *tfl1-18* but not in Col-0 at 10 LDs, and 2544 genes differentially expressed in *tfl1-18* but not in Col-0 at 13 LDs (Fig. S5). Combining these two lists identified 3676 genes differentially expressed in *tfl1-18* but not in Col-0 (1132, 1997 and 547 only differentially expressed at 10 LDs, 13 LDs or in both time points, respectively; Fig. S5). This set of *tfl1*-specific DEGs is enriched for genes directly bound by TFL1 or FD (TFL1 targets *P*=1.21E-15; FD targets *P*=1.45E-23; Fig. S8). For these DEGs, the fold-change between Col-0 and *tfl1-18* was calculated at 7, 10, 13 LDs (e.g. 7 LDs Col-0 versus 7 LDs *tfl1-18*) followed by clustering analysis. Five clusters were identified, and Clusters 1 and 4 contained only one and two genes, respectively, so were not considered further (Fig. S7). Cluster 5 contained 3098 genes that were mildly reduced in expression in *tfl1-18* compared with Col-0, whereas Clusters 2 and 3 contained genes that were increased in expression in the mutant compared with Col-0, and contained 535 and 40 genes, respectively (Fig. 3C; Fig. S7; Table S1). Because TFL1 is considered a repressor of transcription (Ahn et al., 2006; Hanano and Goto, 2011), we focused our analysis on genes increased in expression in *tfl1-18* compared with Col-0 (Clusters 2 and 3). We observed that genes in Cluster 2 were mildly increased in expression at 10 LDs and more strongly increased at 13 LDs compared with Col-0, with a log_2_(fold change) of ∼1 at 13 LDs (Fig. S7). This cluster is enriched for genes in the gene ontology (GO) category of flower development, and in subcategories that are associated with regulation of the floral transition, such as positive regulation of flower development, positive regulation of reproductive process and response to gibberellin (Fig. S7). It is also enriched for TFL1 and FD targets based on ChIP-seq data (TFL1 targets *P*=2.89E-05, FD targets *P*=2.13E-09) (Fig. S8; Table S1) (Collani et al., 2019; Goretti et al., 2020; Romera-Branchat et al., 2020; Zhu et al., 2020). Examples of genes in Cluster 2 are *FRUITFULL* (*FUL*), *SQUAMOSA PROMOTER BINDING PROTEIN-LIKE 3* (*SPL3*), *SPL5*, *SPL8* and *ETHYLENE RESPONSE FACTOR 12* (*ERF12*) (Fig. 3C,D; Table S1; Fig. S9). The earlier increase in expression of these genes in *tfl1-18* compared with Col-0 is consistent with the early-flowering phenotype of the mutant. In contrast, Cluster 3 contained genes that show a high increase in expression in *tfl1-18* already at 10 LDs as well as at 13 LDs, where the average log_2_(fold change) is approximately 4 and 2, respectively, compared with Col-0 (Fig. S7). Cluster 3 is also enriched for genes in the GO category of flower development, but in subcategories not enriched in Cluster 2, such as floral meristem growth, floral meristem identity and floral organ maintenance (Fig. S7). It is also enriched for TFL1 and FD targets (TFL1 targets *P*=0.003, FD targets *P*=0.017) (Fig. S8; Table S1) (Collani et al., 2019; Goretti et al., 2020; Romera-Branchat et al., 2020; Zhu et al., 2020). Examples of genes in Cluster 3 are *AP1*, *SEP3*, *AP3* and *AGAMOUS* (*AG*) (Fig. 3C,D; Table S1; Fig. S9).

Whether these clusters behave similarly under SD conditions was tested (Fig. S10). Initially, the morphology of SAMs of Col-0 plants and *tfl1-18* mutants grown under SDs were examined, and in both genotypes floral primordia were first detected 6 weeks after germination (Fig. S10C). Accordingly, selected genes of Cluster 2 (*FUL*, *SEP4*, *SPL3* and *SPL8*; Fig. S10A) showed no differences in their temporal expression patterns between the two genotypes. However, at 8 weeks SD, several genes of Cluster 3 were more highly expressed in *tfl1* than in Col-0 (*AP1*, *SEP1*, *SEP2*, *AP3*; Fig. S10B) and at 10 weeks SD a terminal flower was formed in *tfl1-18* plants. Therefore, under SDs no difference in Cluster 2 gene expression was detected between *tfl1-18* and Col-0, correlating with their similar flowering time under these conditions, but several Cluster 3 genes increased in expression as *tfl1-18* proceeded to form a terminal flower.

Taken together, TFL1 acts early after germination under LDs and later during vegetative development under SDs to completely block the expression of Cluster 3 genes that are expressed in flowers of Col-0 and contribute to floral development. By contrast, at the same time points under LDs, TFL1 weakly reduces the expression of Cluster 2 genes that promote floral transition. Under SDs, these genes are not detectably regulated by TFL1. Both clusters are enriched in genes directly bound by FD or TFL1. This is consistent with the hypothesis that during the early stages of seedling development under LDs, TFL1 blocks floral development while only modulating the time of floral transition.

### The spatial and temporal expression patterns of genes in Clusters 2 and 3 confirm different modes of gene regulation by TFL1

**Fig. 4.**
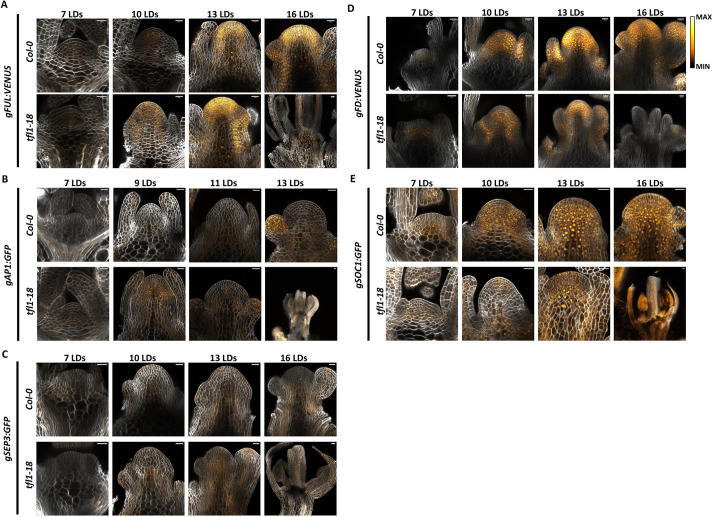
**Spatio-temporal expression patterns in *tfl1-18* and Col-0.** (A-E) Confocal images of FUL:VENUS (A), AP1:GFP (B), SEP3:GFP (C), FD:VENUS (D) and SOC1:GFP (E) in *tfl1-18* and Col-0 at 7, 10, 13 and 16 LDs. Scale bars: 20 µm.

On the basis of the RNA-seq results, the temporal and spatial expression patterns of several flowering-related genes were investigated in more detail by confocal microscopy and *in situ* hybridisation in Col-0 and *tfl1-18* apices. The spatial patterns of FUL protein, a member of Cluster 2 (Fig. 3C), and AP1 and SEP3 proteins, members of Cluster 3 (Fig. 3C), were analysed by confocal microscopy using translational fusions to fluorescent proteins. In agreement with the RNA-seq data, FUL-VENUS is detected throughout the SAM earlier in *tfl1-18* than in Col-0 (10 LDs and 13 LDs, respectively), such that the protein appears in both genotypes at a similar developmental stage when the meristem domes (Fig. 4A). These data support the hypothesis that in Col-0, *FUL* is repressed by TFL1 during the vegetative phase and enhanced during doming when TFL1 protein abundance is distributed more homogeneously and not concentrated in subdomains of the SAM (Fig. 2). In *tfl1-18*, the mRNA of the Cluster 3 gene *AP1* was previously shown to be mislocalised in the inflorescence meristem (Gustafson-Brown et al., 1994) (Fig. S11), but when the gene is first misexpressed in the SAM has not been systematically examined. In our analysis, AP1-GFP was found to be expressed only after floral transition in Col-0 and localised to floral buds as expected, but was detected in the centre of the SAM in *tfl1-18* at 10 LDs during the doming stage, before floral development (Fig. 4B). *SEP3* was also assigned to Cluster 3, and in *tfl1-18*, SEP3-GFP was localised at 10 LDs in the centre of the SAM (Fig. 4C). However, in Col-0, SEP3-GFP was not detected in the SAM but was present in the floral meristem, as previously reported for *SEP3* mRNA (Mandel and Yanofsky, 1998). These results show that expression of the Cluster 3 genes *AP1* and *SEP3* is temporally and spatially mislocalised in *tfl1-18* so that they are expressed in the SAM at an early stage of floral transition during doming.

**Fig. 5.**
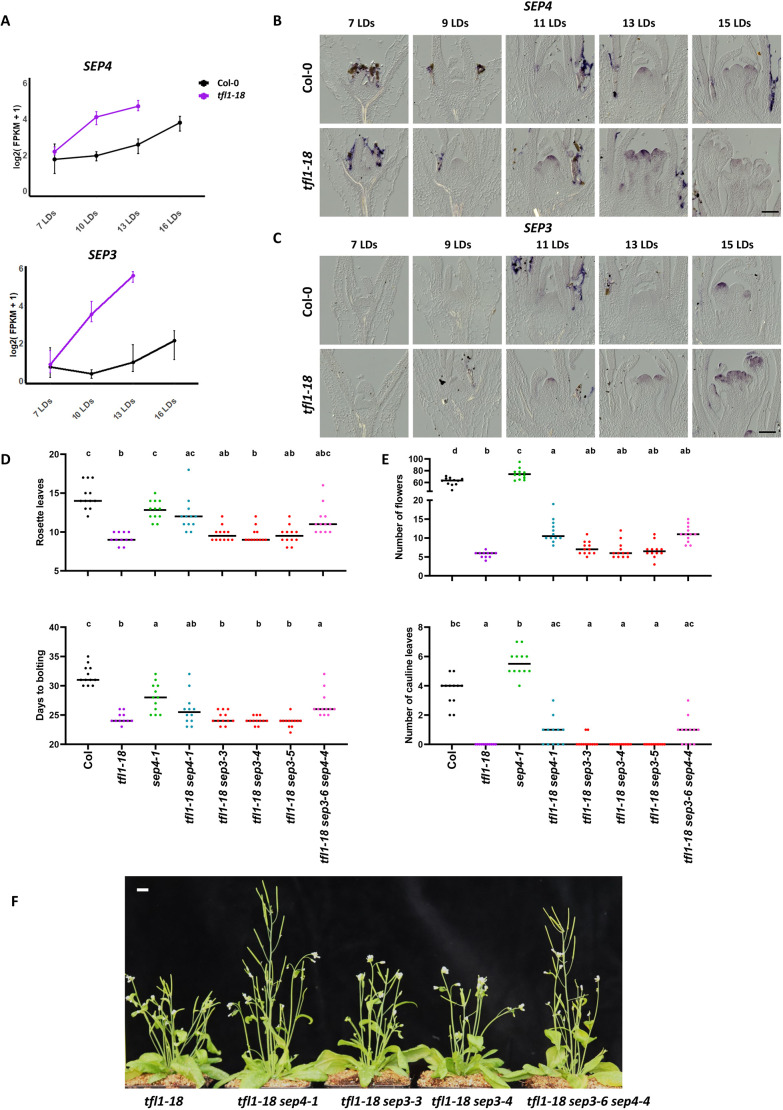
**SEP3 and SEP4 analysis in *tfl1-18*.** (A) Expression of *SEP3* and *SEP4* in Col-0 and *tfl1-18* based on RNA-seq reads. (B) *In situ* hybridisation of *SEP4* in Col-0 and *tfl1-18* during floral transition. (C) *In situ* hybridisation of *SEP3* in Col-0 and *tfl1-18* during floral transition. (D) Number of rosette leaves and days to bolting of Col-0, *tfl1*, *sep4*, *tfl1 sep4*, *tfl1 sep3* and *tfl1 sep3 sep4*. (E) Number of siliques at the end of inflorescence development and number of cauline leaves of Col-0, *tfl1*, *sep4*, *tfl1 sep4*, *tfl1 sep3* and *tfl1 sep3 sep4*. For normally distributed data, one-way ANOVA followed by Tukey's multiple comparisons test was used to test means; for non-normal distributions, the Kruskal–Wallis test followed by Dunn's multiple comparisons test was used. Lowercase letters are used to label means, such that bars bearing different letters are statistically different from one another with a minimum *P*-value of <0.05. Horizontal bar shows mean. (F) Picture of *tfl1*, *tfl1 sep4*, *tfl1 sep3* and *tfl1 sep3 sep4* plants at the end of inflorescence development. Scale bars: 100 µm (B,C); 1 cm (F).

To extend the analysis of DEGs in *tfl1-18*, we focused on the SEP MADS-box proteins, which act redundantly in conferring floral organ identity (Ditta et al., 2004; Goto, 2001; Pelaz et al., 2000), but in our analysis *SEP1*, *SEP2* and *SEP3* appear in Cluster 3, whereas *SEP4* is in Cluster 2, suggesting a role for SEP4 both in the regulation of floral transition and inflorescence meristem maintenance. Based on their expression profiles in the RNA-seq data, *SEP4* and *SEP3* are the most differentially expressed genes in this family (Fig. 5A; Fig. S13). By *in situ* hybridisation, *SEP4* mRNA is first detected in the inflorescence meristem of Col-0 at doming (13 LDs) and in the inflorescence is detected in floral primordia (Fig. 5B). In *tfl1-18*, *SEP4* mRNA appears earlier, at 9 LDs, when doming occurs and continues to be detected more strongly than in Col-0 (Fig. 5B). *SEP1*, *SEP2* and *SEP3* mRNAs were not detected in the Col-0 meristem before, during or after floral transition and were expressed only in floral primordia (Fig. 5C; Fig. S13). However, the three genes are all ectopically expressed in the SAM of *tfl1-18* from approximately day 11 (Fig. 5C; Fig. S13). This analysis further illustrates that the Cluster 2 gene *SEP4*, as described earlier for *FUL*, is expressed earlier and at higher levels in the SAM in the *tfl1-18* mutant compared with Col-0, whereas expression of Cluster 3 genes (*SEP1*, *SEP2* and *SEP3*, as described for *AP1*) is not detected in the SAM of Col-0, but they are ectopically expressed at early stages of development in the SAM of *tfl1-18*.

As a control, we also analysed the expression profile of *FD* and *SOC1*, which are important regulators of floral transition (Abe et al., 2005; Onouchi et al., 2000; Wigge et al., 2005) but are not present in Clusters 2 and 3 and do not change in *tfl1-18* with respect to Col-0. In agreement with the RNA-seq, the localisation of FD:VENUS (Romera-Branchat et al., 2020) was similar in both genotypes (Fig. 4D). In Col-0, *gSOC1:GFP* was expressed ubiquitously throughout the SAM during floral transition, but not in floral primordia (Fig. 4E). However, in *tfl1-18*, SOC1-GFP was absent from the central region of the meristem at doming (10 LDs) (Fig. 4E). In floral buds of Col-0 and in the inflorescence meristem of *tfl1-*18, the expression domains of SOC1 and AP1 were complementary (Fig. 4B,E; Fig. S11), suggesting that the lack of SOC1-GFP in the centre of the *tfl1-18* meristem might be a consequence of the misregulation of AP1 and other Cluster 3 proteins. However, *gFUL:VENUS* expression appears not to be repressed by AP1 in the inflorescence SAM (Fig. 4A). Therefore, the misexpression in the SAM of genes involved in floral development, such as *AP1*, appears to alter the spatial and temporal expression patterns of some genes involved in the promotion of floral transition or inflorescence development that are normally expressed in the SAM of Col-0 (Fig. S12).

### *SEP4* and *FD* contribute to inflorescence meristem indeterminacy in *tfl1-18* mutants

**Fig. 6.**
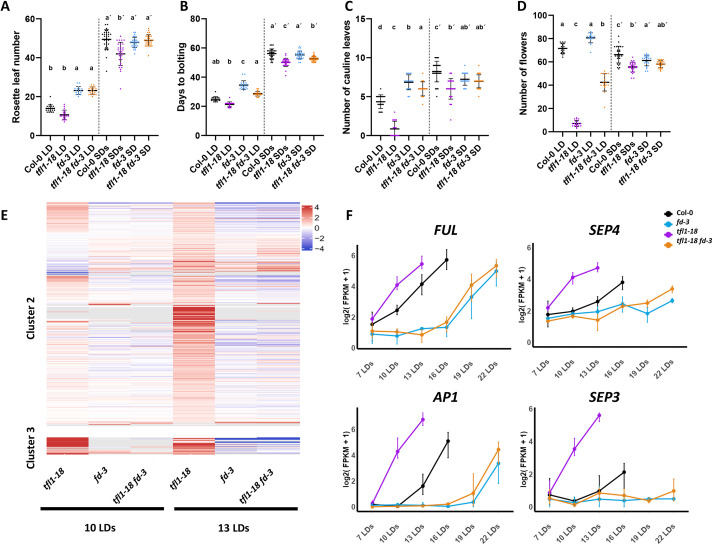
**The *fd* mutation suppresses the effects of *tfl1* on flowering phenotypes and gene expression.** (A-D) Number of rosette leaves (A), days to bolting (B), number of cauline leaves (C) and number of flowers formed on the inflorescence (D) under long-day (LD) and short-day (SD) conditions. For normally distributed data, one-way ANOVA followed by Tukey's multiple comparisons test was used to compare means; for non-normal distributions, the Kruskal–Wallis test followed by Dunn's multiple comparisons test was used. Statistical tests for LD and SD conditions were conducted separately. Lowercase letters are used to label means, such that bars bearing different letters are statistically different from one another with a minimum *P*-value of <0.05. Data are mean±s.d. (E) Heat map showing the expression of genes of Cluster 2 and Cluster 3 in Col-0 versus *tfl1-18*, *fd-3* and *tfl1-18 fd-3* at 10 and 13 LDs. (F) Expression of *FUL*, *SEP4*, *AP1* and *SEP3* in Col-0, *tfl1-18*, *fd-3* and *tfl1-18 fd-3* during floral transition based on the RNA-seq data as described in Fig. 3.

To determine how flowering-time genes, including specific DEGs in Clusters 2 and 3, contribute to the *tfl1-18* mutant phenotypes, several double mutant combinations were constructed. Previously, the *fd* mutation was shown to suppress the inflorescence and flowering-time phenotypes of *tfl1* (Hanano and Goto, 2011; Jaeger et al., 2013). We constructed the *tfl1-18 fd-3* double mutant to test the effect of loss of FD activity on all *tfl1-18* phenotypes in our conditions under LDs and SDs. Both *fd-3* and *tfl1-18 fd-3* were late flowering compared with Col-0 under LDs, producing the same number of rosette leaves, although *tfl1-18 fd-3* bolted slightly earlier than *fd-3*. Therefore, with respect to flowering time, the *fd-3* mutation is largely epistatic to *tfl1-18* under LDs (Fig. 6A,B). Under LDs and during the I1 phase of inflorescence development, when cauline leaves and axillary branches are formed, *fd-3* and *tfl1-18* produced more or fewer paraclades than Col-0, respectively, whereas *tfl1-18 fd-3* formed slightly fewer paraclades than *fd-3* but more than Col-0 (Fig. 6C). Therefore, during I1, *fd-3* is largely, but not fully, epistatic to *tfl1-18*. During the I2 phase of inflorescence development, when flowers are formed, *tfl1-18* and *tfl1-18 fd-3* both produced fewer flowers than Col-0, suggesting that FD is less important for TFL1 function during I2 phase than during I1 (Fig. 6D). Under SDs, flowering-time of the *tfl1-18* mutant was delayed compared with that of plants under LDs, as expected (Shannon and Meeks-Wagner, 1991), but plants still flowered significantly earlier than Col-0 and formed fewer cauline leaves and flowers in the inflorescence (Fig. 6A-D). The *fd-3* mutation was also epistatic to *tfl1-18* in rosette leaf number under SDs (Fig. 6A-D). These results are consistent with previous data (Hanano and Goto, 2011; Jaeger et al., 2013), but extend the analysis to SDs and demonstrate that in *tfl1-18* mutants, FD is less critical for I2 development than for rosette leaf and I1 development.

*SEP3* (Cluster 3) and *SEP4* (Cluster 2) are strongly misexpressed in *tfl1-18*; therefore, genetic interactions among *sep4*, *sep3* and *tfl1* were tested. Existing T-DNA insertion alleles and newly induced CRISPR alleles were used (Fig. S15). The *sep4-1 tfl1-18* double mutants produced significantly more rosette leaves and I2 nodes than *tfl1-18* mutants and often formed more I1 nodes, although this was not statistically significant (Fig. 5). Therefore, the *SEP4* gene from Cluster 2 makes a significant contribution to the flowering time and inflorescence phenotypes of *tfl1-18*. Furthermore, *sep4-1* mutants formed a similar number of rosette and cauline leaves to Col-0, but bolted earlier and produced more flowers (Fig. 5D,E). The triple mutant *tfl1-18 sep3-6 sep4-4* also produced more nodes during I2 development than *tfl1-18*, but a similar number to *tfl1-18 sep4-1*, suggesting that SEP3 and SEP4 are not redundant for these activities. Consistent with this conclusion, mutation of *SEP3* alone did not influence the *tfl1-18* phenotype significantly during vegetative, I1 or I2 phases (Fig. 5), despite being strongly misexpressed from an early stage during vegetative development. In contrast to the role of *SEP4* in *tfl1-18*, mutations in a second Cluster 2 gene, *FUL*, or the related MADS-box gene *SOC1*, did not significantly affect any aspect of the *tfl1-18* phenotype (Fig. S14). Taken together, these data suggest that, in addition to FD, the Cluster 2 gene *SEP4* plays a significant role in the *tfl1-18* mutant phenotype during vegetative and inflorescence development.

### Time-resolved transcriptomic analysis during floral transition indicates that almost all gene misexpression in *tfl1-18* depends on FD

The *fd-3* mutation largely suppressed the phenotypic effects of *tfl1-18* on vegetative and I1 development (Hanano and Goto, 2011; Jaeger et al., 2013). To determine to what extent FD is required for the genome-wide effects of TFL1 on gene expression at the shoot apex before and during floral transition, the time-resolved transcriptomic RNA-seq data of apices of *fd-3* and *tfl1-18 fd-3* were compared with the data already described above for Col-0 and *tfl1-18* (Table S4). To investigate whether genes of Clusters 2 and 3 were activated in *tfl1-18* background in the absence of FD, the expression of these genes in Col-0 was compared at 10 LDs and 13 LDs with *tfl1-18*, *fd-3* and *tfl1-18 fd-3* (Fig. 6E). We observed that the absence of *FD* was sufficient to downregulate the expression of almost all Cluster 2 and 3 genes at 10 and 13 LDs in *tfl1-18* background, which correlated with the delayed flowering time and extended inflorescence development of *tfl1-18 fd-3* compared with *tfl1-18*. The expression of *SEP4*, *FUL*, *SEP3* and *AP1* were analysed in these data (Fig. 6F) and were found to increase in expression much later in *tfl1-18 fd-3* mutants compared with *tfl1-18*, consistent with a direct role of FD in their activation (Fig. 6F). These data suggest that FD, probably through FT signalling, increases the expression of almost all Cluster 2 and 3 genes during the early stages of floral transition of *tfl1* mutants.

Overexpression of FT can confer a terminal flower phenotype due to the antagonistic functions of FT or TFL1 (Kobayashi et al., 1999). To test whether increased *FT* transcription could contribute to the *tfl1-18* phenotype, the levels of *FT* mRNA and of genes related to *FT* activity were measured in leaves of Col-0 and *tfl1-18* plants at different times under LDs, and in the RNA-seq data of apices of LD-grown plants (Fig. S16). The levels of *GIGANTEA* (*GI*), *CONSTANS* (*CO*), *FT*, *FD* and *SOC1* mRNAs were not increased in leaves of *tfl1-18* compared with Col-0, supporting the hypothesis that TFL1 does not repress the transcription of these genes in leaves, but rather reduces sensitivity to FT signalling at the shoot apex (Fig. S16). In apices, *FT* mRNA was not increased in *tfl1-18*, but *FD* mRNA level was higher at 10 LDs, and could contribute to enhanced sensitivity to the FT signal in *tfl1-18* mutants.

## DISCUSSION

In *Arabidopsis*, *TFL1* has a dual role in determining plant architecture (Benlloch et al., 2015; McGarry and Ayre, 2012). First, it represses the transition from vegetative growth to I1 development, increasing the number of rosette leaves formed (Bradley et al., 1997; Shannon and Meeks-Wagner, 1991). Second, it prevents the formation of a terminal flower, thereby maintaining inflorescence meristem indeterminacy so that more I1 and I2 nodes are formed (Bradley et al., 1997; Shannon and Meeks-Wagner, 1991). Similarly, in other species *TFL1* orthologues show related functions that can be influenced by the developmental programme of the inflorescence (e.g. monopodial or sympodial) and by the life history of the plant (e.g. annual or perennial) (Benlloch et al., 2015; Perilleux et al., 2019). In some species, such as *Antirrhinum majus*, the TFL1 orthologue maintains the indeterminacy of the inflorescence but does not delay floral transition. By contrast, in pea (*Pisum sativum*) the dual function of *TFL1* diverged between two paralogues, one of which delays floral transition while the other maintains inflorescence indeterminacy (Benlloch et al., 2015; Foucher et al., 2003).

By quantification of confocal microscope images, we found that the two roles of *TFL1* in Arabidopsis correlate with distinct patterns of TFL1 accumulation at the shoot apex. Indeed, TFL1 accumulates below the meristem dome during the vegetative phase, but at the tip of the SAM during inflorescence development. Examination of meristem morphology and analysis of transcriptomes demonstrated that *tfl1-18* mutants deviate from Col-0 wild type at an early stage of floral transition under LDs. The SAM of the *tfl1-18* mutant already domes around 10 days after germination and genes involved in promotion of reproduction (Cluster 2) gradually rise in expression at the same stage, three days earlier than in Col-0. In parallel, a second cluster of genes (Cluster 3) enriched in genes involved in floral meristem identity and floral organ identity is highly expressed in the SAM of *tfl1-18* from 10 LDs and is never expressed in the SAM of Col-0. These analyses indicate that TFL1 contributes to two modes of gene regulation in the wild-type SAM: it modulates the rate of increase in transcription of floral transition genes, and it entirely blocks the transcription of floral organ identity genes. Both of these clusters are enriched in direct target genes of TFL1 and FD, and their misexpression in *tfl1* mutants depends upon FD activity. We propose that the dynamic pattern of TFL1 accumulation and its different modes of gene repression shape the local antagonism between TFL1 and FT in the SAM that is proposed to regulate floral transition and inflorescence development (Kardailsky et al., 1999; Kobayashi et al., 1999; McGarry and Ayre, 2012; Shalit et al., 2009).

### TFL1 delays floral transition and Cluster 2 gene expression by repressing FD activity

During floral transition, the first function of *TFL1* is to delay inflorescence meristem establishment under LDs. Accordingly, Cluster 2 is enriched for genes that promote the transition to reproductive development, which are progressively activated in the SAM of Col-0 and are expressed at higher levels in *tfl1* than in Col-0 early during floral transition. Moreover, we found that in *tfl1*, the higher and earlier expression level of the majority of these genes is dependent on FD. Therefore, in Col-0, TFL1 likely reduces the capacity of FD to activate transcription of Cluster 2 genes in the SAM and thereby delays flowering. Furthermore, direct targets of TFL1 or FD are enriched among the Cluster 2 DEGs, suggesting that a complex of TFL1–FD may directly repress transcription of these genes early in development (Collani et al., 2019; Goretti et al., 2020; Romera-Branchat et al., 2020; Zhu et al., 2020). However, in *tfl1-18* mutants and later in development of Col-0, their activation occurs in the SAM via FD, perhaps through interaction with FT (Abe et al., 2005; Hanano and Goto, 2011; Wigge et al., 2005). This suggestion was supported by the analysis of the expression of Cluster 2 genes under SD conditions, where FT does not control flowering time and no difference in expression level was detected for these genes between *tfl1-18* and Col-0. Finally, mutation of one of the Cluster 2 genes, *SEP4*, delays flowering of *tfl1-18*, supporting the role of these genes in promoting floral transition in *tfl1* mutants.

The proposed gradual transition in wild-type Col-0 from repression of Cluster 2 genes by TFL1–FD to their activation by FT–FD correlates with an alteration in the pattern of TFL1 accumulation in the SAM. At the doming stage, when the expression levels of Cluster 2 genes rise in Col-0, TFL1-VENUS is transiently present in a broad diffuse pattern. This transient pattern occurs in a short interval between the vegetative stage, when TFL1 accumulates narrowly below the meristem, and the inflorescence stage, when TFL1 accumulates at the tip of the meristem. Nevertheless, *TFL1* mRNA was not detected at the tip of the inflorescence meristem where the protein accumulates, consistent with movement of the protein from the cells in which *TFL1* is transcribed (Conti and Bradley, 2007; Goretti et al., 2020). Similarly, a transcriptional fusion of *TFL1* to the marker gene encoding β-glucuronidase was expressed in a broader pattern at the transition stage around doming than in the vegetative meristem (Serrano-Mislata et al., 2016), and in *Arabis alpina*, a relative of *A. thaliana*, a dynamic pattern of transcription of the *TFL1* orthologue was detected during floral induction in response to vernalisation (Wang et al., 2011). The increased and apical expression of *TFL1* mRNA in the inflorescence meristem of *Arabidopsis* is probably due to activation of its transcription by factors induced in the SAM during floral induction, such as SOC1, AGL24 and XAL2 (Azpeitia et al., 2021; Perez-Ruiz et al., 2015; Serrano-Mislata et al., 2016), but how the pattern of *TFL1* transcription below the meristem during vegetative development is controlled remains unclear. We observe that during the transient doming phase between vegetative and inflorescence development there is a time interval during which TFL1 accumulation is more diffuse and presumably lower in concentration in the SAM (Fig. S4B). This pattern may be a consequence of the SAM increasing in size and doming during floral transition, while *TFL1* transcription still occurs at relatively low levels only in the cells below the SAM where it is expressed during vegetative development (Fig. S2) (Bradley et al., 1996; Kinoshita et al., 2020). We propose that this transient diffuse pattern may reduce the ability of TFL1 to antagonise FT at the SAM, enhancing the activation of transcription of Cluster 2 genes and flowering during the transition stage. This hypothesis of temporal and spatial variation in the competition between FT and TFL1 at the SAM should be tested in the future by comparing the spatial accumulation of both proteins in apices during the transition from a vegetative to inflorescence SAM.

### TFL1 maintains indeterminate shoot development by stable repression of floral organ identity genes during early vegetative growth

After floral transition, the inflorescence meristem of *tfl1* mutants rapidly develops into a terminal flower greatly reducing the number of I1 and I2 nodes compared with Col-0. We found that Cluster 3 genes, which are highly expressed in the SAM of 10-day-old seedlings and never in the SAM of Col-0, are enriched for those in the GO category floral development, and include *AP1* and *AG* as well as others in the GO categories associated with floral organ and floral meristem maintenance. The expression of these genes in the SAM of *tfl1-18* early during floral transition depends upon FD and they are enriched for direct targets of FD and TFL1. These observations suggest that in Col-0, the TFL1–FD complex mediates the repression of these genes within the SAM and that this is not overcome in the SAM during floral induction by FT–FD. Therefore, when Cluster 2 genes rise in expression in the Col-0 SAM during doming, Cluster 3 genes remain repressed. This difference in regulation may indicate that Cluster 3 genes are more sensitive to TFL1-mediated transcriptional repression, and only become expressed in mature flowers where TFL1 is entirely absent. Alternatively, in Col-0, TFL1–FD may directly repress Cluster 3 genes at early stages of development, and then in the domed meristem as TFL1 concentration is reduced and Cluster 2 genes are expressed, Cluster 3 genes may be repressed by a secondary mechanism that involves transcription factors expressed during floral transition. In the mature inflorescence meristem, TFL1 protein accumulates at the tip of the SAM and therefore the TFL1–FD complex could again stably repress Cluster 3 floral development genes at this stage. However, Cluster 3 genes are repressed in the SAM, their ectopic activation in young *tfl1-18* mutants clearly relies on FD activity and may involve SEP4, as *tfl1-18 sep4-1* double mutants are delayed in the formation of a terminal flower, partially rescuing the plant architecture defect observed in *tfl1-18*.

### Distinct contributions of *SEP4* and *SEP3* to the *tfl1* mutant phenotype

We found that all four SEP genes are differentially expressed in *tfl1-18* with respect to Col-0. The MADS-box transcription factors encoded by these genes share high homology and contribute to the function of many tetrameric complexes during flower development (Ditta et al., 2004; Goto, 2001; Pelaz et al., 2000; Theissen and Saedler, 2001). Although several members of the MADS-box family, such as FUL and SOC1, contribute to floral transition and interact with SEP3 and SEP4 (de Folter et al., 2005), the role of SEP proteins in flowering time has not been extensively studied. *SEP3* and *SEP4* were the most deregulated SEP genes in *tfl1-18*, and therefore we generated double and triple mutants combining *tfl1-18* with *sep3* or *sep4* mutations to test their effects on the early flowering and inflorescence phenotypes of *tfl1-18*. Analysis by RNA-seq, *in situ* hybridisation and confocal microscopy placed *SEP4* and *SEP3* in Cluster 2 and Cluster 3, respectively. Moreover, in contrast to *SEP3*, *SEP4* is expressed in the SAM of Col-0 during floral transition. This suggests that SEP4 regulates floral transition progression in addition to flower development and highlights differences in *SEP4* and *SEP3* transcriptional regulation during development. Indeed, mutation of *SEP4* in *tfl1-18* delays bolting and increases the number of I1 and I2 nodes formed before SAM termination. By contrast, despite being ectopically expressed in the SAM from 10 LDs of *tfl1-18*, *SEP3* alone does not appear to influence flowering time nor indeterminacy. Protein interaction networks previously compiled based on yeast two-hybrid analysis implicated SEP1, SEP2 and SEP3 more widely in regulatory complexes involved in floral development than SEP4, whereas all SEP proteins interacted with MADS-box proteins involved in flowering time (de Folter et al., 2005). Our data indicate that, although SEP3 and SEP4 are closely related paralogues that show differences in mRNA expression, they likely also have distinct biochemical functions that are revealed genetically by their different effects when misexpressed in the SAM of the *tfl1* mutant. The observation that *sep4* delays both flowering and shoot determinacy of *tfl1-18* suggests that SEP4 contributes to MADS-box complexes promoting flowering time and terminal flower production.

## MATERIALS AND METHODS

### Plant material and growth conditions

All mutants were in the *A. thaliana* ecotype Columbia (Col-0) background: *tfl1-18* (GABI_228A07; Rosso et al., 2003), *fd-3* (SALK_054421; Jang et al., 2009), *ful-2* (Ferrándiz et al., 2000), *soc1-6* (SALK_138131; Immink et al., 2012) and *soc1-2* (Lee et al., 2000), *sep4-1* (Ditta et al., 2004). The *fd-3*, *ful-2*, *sep4-1*, *soc1-6* and *soc1-2* mutants were crossed to *tfl1-18* to obtain double mutants. The following transgenic lines were used: *gFD:VENUS* and *gFDP:VENUS* (Romera-Branchat et al., 2020), *gSOC1:GFP* (Immink et al., 2012), *gFUL:VENUS* (Driel, 2021), *gAP1:GFP* (Urbanus et al., 2009), *gSEP3:GFP* (de Folter et al., 2007) and *pFD:3xHA-mCHERRY-FD* (Martignago et al., 2023). Plants were grown on soil under controlled conditions of SD (8 h light/16 h dark) or LD (16 h light/8 h dark) at 18-22°C and a light intensity of 150-175 μmol m^−2^ s^−1^.

### Marker lines cloning

Cloning of the *TFL1* locus was based on polymerase incomplete primer extension (Klock and Lesley, 2009). All PCR amplifications were performed with Phusion Enzyme (New England Biolabs). Primers TF01-F and TF02-R were used to amplify the *TFL1* genomic region (7.8 kb) and the PCR product was cloned into *pDONR207* by BP reaction. The primer pairs TF014 and TF016, were used to amplify linker-VENUS (0.7 kb). The primers TF014-F and TF015-R were used to linearise the construct *TFL1-pDONR207* and the linker-VENUS amplicon was added to construct the plasmid *pTFL1:TFL1:VENUS:tTFL1-pDONR207* via Gibson assembly. Subsequently, the plasmid was cloned into the binary vector pEarleyGate301 (Earley et al., 2006) by LR reaction. All primers used are listed in Table S2.

### CRISPR cloning

Four gRNA sequences were designed using the CCTop website to target *SEP3* and *SEP4* (Fig. S15) (Labuhn et al., 2018; Stemmer et al., 2015). A DNA fragment containing the specific guides, the DNA scaffold and t-RNA sequence was synthesised by Thermo Fisher Scientific (Fig. S15). Enzymatic digestion with *Aar*I followed by ligation was used to insert the synthesised fragment into the pKIR1 plasmid (Fig. S15) (Tsutsui and Higashiyama, 2017). Later, *tfl1-18* plants grown under SD were transformed via floral dipping. The transgenic seeds were selected under a fluorescence microscope. *SEP3* and *SEP4* were amplified by PCR and potential new alleles were identified by Sanger sequencing (Fig. S15).

### Quantification of SAM size and MorphoGraphX

For confocal acquisition, apices were harvested and fixed in 4% paraformaldehyde by vacuum infiltration twice for 10 min. Samples were incubated overnight in the dark, washed twice with 1× PBS buffer and immersed in ClearSee solution for 3-10 days (Kurihara et al., 2015). Two days before image acquisition, samples were stained with Renaissance 2200 [0.1% (v/v) in ClearSee] in the dark (Musielak et al., 2015). The width and height of the meristem were measured from a confocal image of a sagittal section of the meristem centre with Fiji software (Schindelin et al., 2012). The width of the meristem was measured as a horizontal line from the axil of the youngest primordium flanking the shoot apex to the opposite side of the meristem. Meristem height was measured as the distance from the central tip of the meristem surface perpendicular to the width (see Fig. S1). For MorphoGraphX acquisition, samples were mounted on slides in low-melting agarose and imaged with a Leica SP8 confocal microscope and a PMT detector at an excitation wavelength (λ_ex_) of 405 nm and emission wavelength (λ_em_) of 450-470 nm. The objective lens was HC PL APO CS2 40×/1.25 GLYC (Leica) and the *z*-step interval was 0.3 µm. MorphoGraphX analysis was performed as described in Kinoshita et al. (2020). The number of biological replicates performed are listed in Table S3.

### Confocal acquisition and quantification of gTFL1:VENUS and gFD:mCHERRY

Apices were harvested and prepared for confocal imaging as described above. Each meristem was mounted on its side in ClearSee and covered by a cover slip. Image acquisition was performed using a Leica Stellaris 5 confocal microscope with HC PL APO 40×/1.25 GLYC motCORR CS2 objective. The parameters used for image acquisition of the fluorophores were: Renaissance 2200, λ_ex_ of 405 nm and λ_em_ of 450-470 nm with Power HyD detector; VENUS, λ_ex_ of 515 nm and λ_em_ of 520-540 nm with a Power HyD detector; mCHERRY, λ_ex_ of 587 nm and λ_em_ of 594-625 nm with a Power HyD detector. The same parameters were used for each acquisition of VENUS and mCHERRY fluorophores. The *xy* resolution was 800×800 pixels, the laser speed 600 Hz and the signal was averaged three times. The entire meristem was imaged using *z*-steps of 0.4 µm, in 16 bits. The number of biological replicates performed are listed in Table S3.

### Quantitative analysis of confocal fluorescence images

Confocal fluorescence *z*-stacks were processed using Matlab custom made code (https://gitlab.com/slcu/teamHJ/pau/RegionsAnalysis/-/releases/TFL1_FD_code). The main goal of this analysis was to extract reproducible measures of fluorescence intensity within the SAM. Fluorescence signal in SAM boundaries was excluded or minimised using a 3D paraboloid mask constructed based on the meristem curvatures in the orthogonal planes. In the sum-of-the-slices projection, a vertical intensity profile that initiated at the SAM apex was computed for each meristem. A smoothing (robust, quadratic) filter was applied to the intensity profiles. The position of the highest intensity peak was identified (position of the maximum). To allow a systematic analysis, the beginning and the end of the expression domains along the SAM longitudinal axis were set to represent the intensity profile points at which 10% of the maximum intensity was reached – or if the intensity level did not decrease below the 10%, then the nearest point to this threshold (see Supplementary Materials and Methods, Figs S17-S21 and Tables S5-S10 for further details). The total intensity was computed by integrating the fluorescence signal (sum of the intensity values) within the domain.

### Confocal image acquisition for protein expression

Apices were prepared as described above. Each meristem was mounted on its side in ClearSee and covered with a cover slip. For gFD:VENUS, gSOC1::GFP, gFDP:VENUS, gFUL:VENUS, gAP1:GFP and gSEP3:GFP, image acquisitions were performed using a Leica SP8 confocal microscope. The parameters used for image acquisition were: Renaissance, λ_ex_ of 405 nm and λ_em_ of 450-470 nm with PMT; GFP, λ_ex_ of 488 nm and λ_em_ of 500-520 nm; VENUS, λ_ex_ of 514 nm and λ_em_ of 520-540 nm with an HyD detector. For GFP and VENUS, the same parameters were used for each acquisition for the whole time course. The number of biological replicates performed are listed in Table S3.

### *In situ* hybridisation

Plants were grown in LD conditions for 17 days and harvested every 2 days from 7 LDs. *In situ* hybridisation was performed as previously described (Torti et al., 2012). The primers used to synthesise the probes are listed in Table S2.

### RNA extraction and sequencing

Apices were harvested at zeitgeber time (ZT) 2-6 and frozen in liquid nitrogen. RNA was extracted using the RNeasy Mini Kit (Qiagen) and eluted in 40 µl nuclease-free water. DNA contamination was removed using the Turbo DNA-free Kit (Thermo Fisher Scientific) and RNA was concentrated using the RNA Clean & Concentrator kit (Zymo Research). At the Max Planck-Genome-Centre Cologne, RNA-seq libraries were built using poly(A) enrichment and were Illumina sequenced with a HiSeq3000 system to obtain 150-bp, single-end reads for a total of 15 million reads per sample.

### RNA-seq analysis

Adapter sequences and low-quality reads were removed using cutadapt (Martin, 2011) and Trimmomatic (Bolger et al., 2014), respectively. Cleaned reads were used to quantify transcript abundance against the *A. thaliana* Reference Transcript Dataset (AtRTD2) (Zhang et al., 2017) using Salmon (Patro et al., 2017). DESeq2 (Love et al., 2014) was employed to transform transcript expression values to the gene level and perform cross-sample normalisation. FPKM values were obtained using the fpkm function of DESeq2 and were transformed by log_2_(FPKM+1). Principal component analysis was performed on the log-transformed FPKM values using the prcomp function of R (https://www.r-project.org/). Pairwise differential expression analyses were performed using DESeq2.

### Gene clustering and GO enrichment tests

Pairwise differential expression values were clustered using the hclust function, and heatmaps were generated and visually inspected to obtain gene clusters using R. The expression pattern of each gene cluster was also visualised as a line plot in which the mean expression was calculated and plotted using R. A GO term enrichment analysis was conducted for Clusters 2 and 3 (Fig. 3) using the Cytoscape Plugin BiNGO (Maere et al., 2005). To perform these analyses, the background consisted of all GO terms annotated to *A*. *thaliana* genes (https://geneontology.org/, January 2021) and the reference set was represented by all genes expressed in the RNA-seq dataset. REVIGO was used to remove highly redundant GO terms using the default settings (Supek et al., 2011). The results were displayed as heatmaps with a purple colour scale reflecting the *P*-value (*P*≤0.05). The ratio of bound to non-bound genes in the DEG set was compared with the same ratio in the non-DEG set using Fisher's exact test implemented in R with the alternate hypothesis set to ‘greater’.

### Real-time PCR

RNA was extracted using the NucleoZOL protocol (Macherey-Nagel) from leaves or apices at different ZTs according to the experimental design and then treated with the Turbo DNA-free Kit (Thermo Fisher Scientific). The cDNA was prepared with 500-1000 ng DNase-treated RNA using the SuperScript IV System (Thermo Fisher Scientific). RT-PCR was performed using GoTaq qPCR MasterMix (Promega A6001) and the primers are listed in Table S2.

## Supplementary Material

10.1242/develop.202089_sup1 Supplementary information

Table S1. RNA-sequencing analysis.

Table S2. Primers sequences and PCR conditions.

Table S3. Number of replicates for confocal acquisition.

Table S4. RNA-sequencing data tables. This data table is available in Dryad repository in the “Fig.3 folder”.
