# Supplementary material for: Two modes of gene regulation by TFL1 mediate its dual function in flowering time and shoot determinacy of Arabidopsis
Source: Development. 2023 Dec 7;150(23):dev202089. doi: 10.1242/dev.202089 (PMC10730086; doi:10.1242/dev.202089)
Supplement: Supplementary information [file develop-150-202089-s1.pdf]

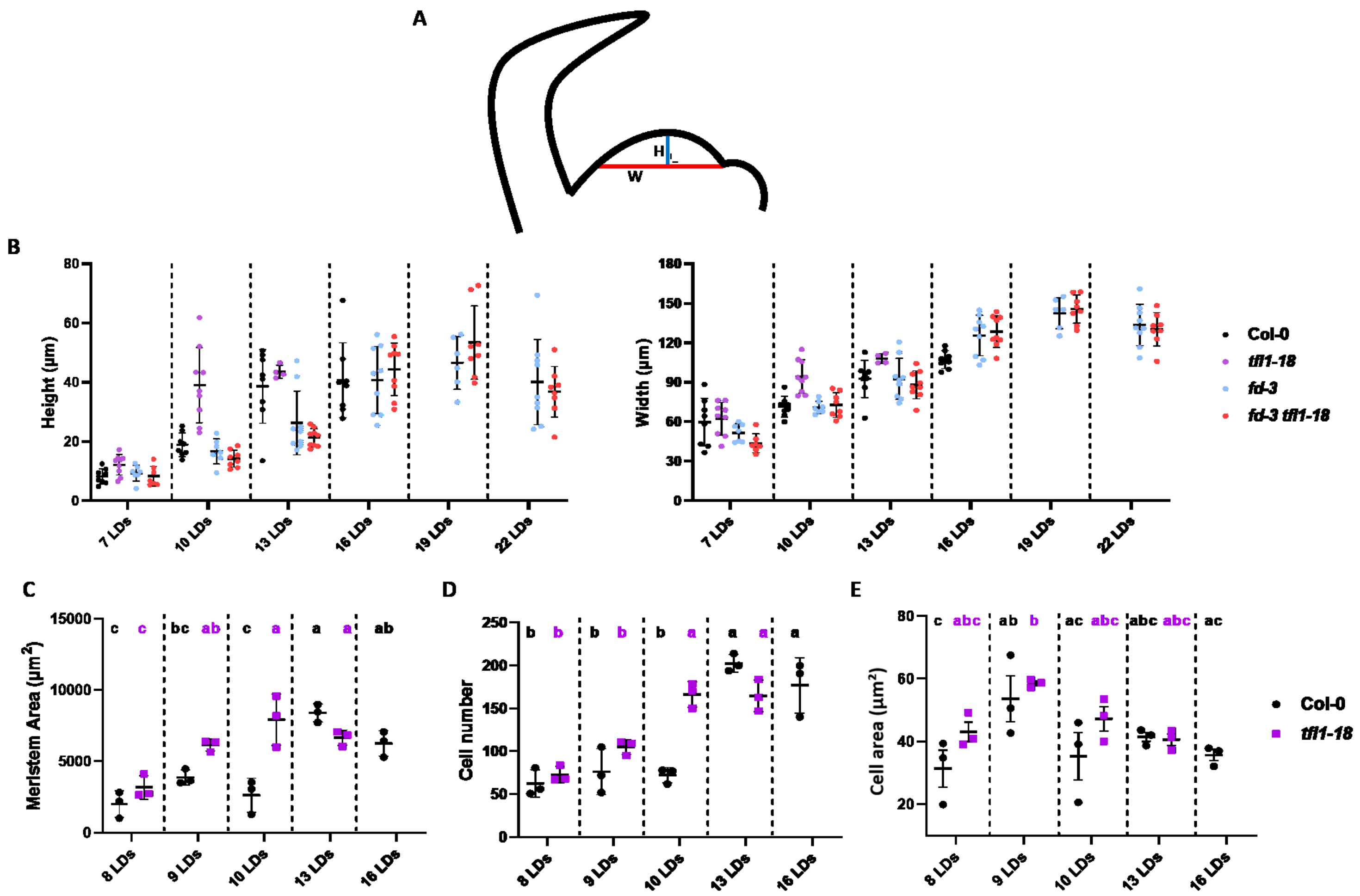

Fig. S1. Analysis of meristem morphology of *tfl1*, *fd*, *fd tfl1* during floral transition.

A) Scheme of the method used to calculate meristem width (red line) and height (blue line). From longitudinal sections through the centre of the meristem, the width was measured from the axil of the most recently formed primordium visible in the section to the opposite side of the meristem in a horizontal line. The height was measured from the tip of the meristem perpendicular to the width.

B) Meristem shape measured by the height and width of Col-0 and *tfl1-18*, *fd-3*, *fd-3 tfl1-18* during floral transition.

C) Meristem area during floral transition of Col-0 and *tfl1-18* calculated using MorphoGraphX software.

D) Number of L1 meristem cells during floral transition of Col-0 and *tfl1-18* calculated using MorphoGraphX software.

E) Area of L1 meristem cells during floral transition of Col-0 and *tfl1-18* calculated using MorphoGraphX software.

To compare means for normally distributed data, ANOVA followed by Tukey´s multiple comparisons test was used. For non-normal distributions, the Kruskal-Wallis test followed by Dunn´s multiple comparisons test was used.

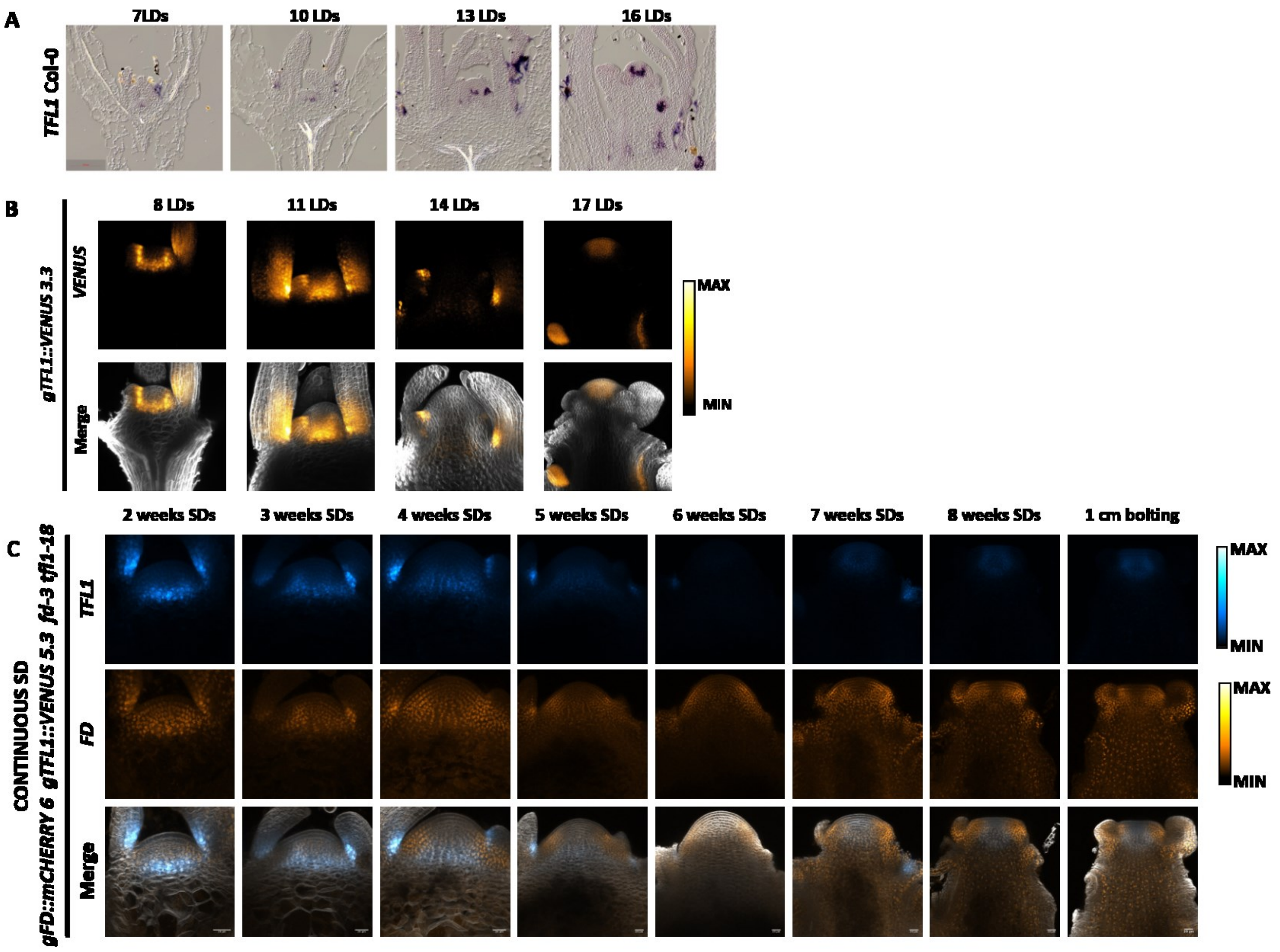

Fig. S2. TFL1 expression and protein localization under LDs and SDs condition

- **A)** *In situ* hybridization of *TFL1* in Col-0 during floral transition.
- **B)** TFL1-VENUS (in orange hot) localization during floral transition in the shoot apical meristem under long-day (LD) conditions. *gTFL1:VENUS* line 3.3 was used.
- **C)** TFL1-VENUS (in cyan hot) and FD-mCHERRY (in orange hot) localization in the shoot apical meristem under short-day (SD) conditions. *gTFL1:VENUS* line 5.3 and *gFD:mCHERRY* line 6 were used.

Development • Supplementary information

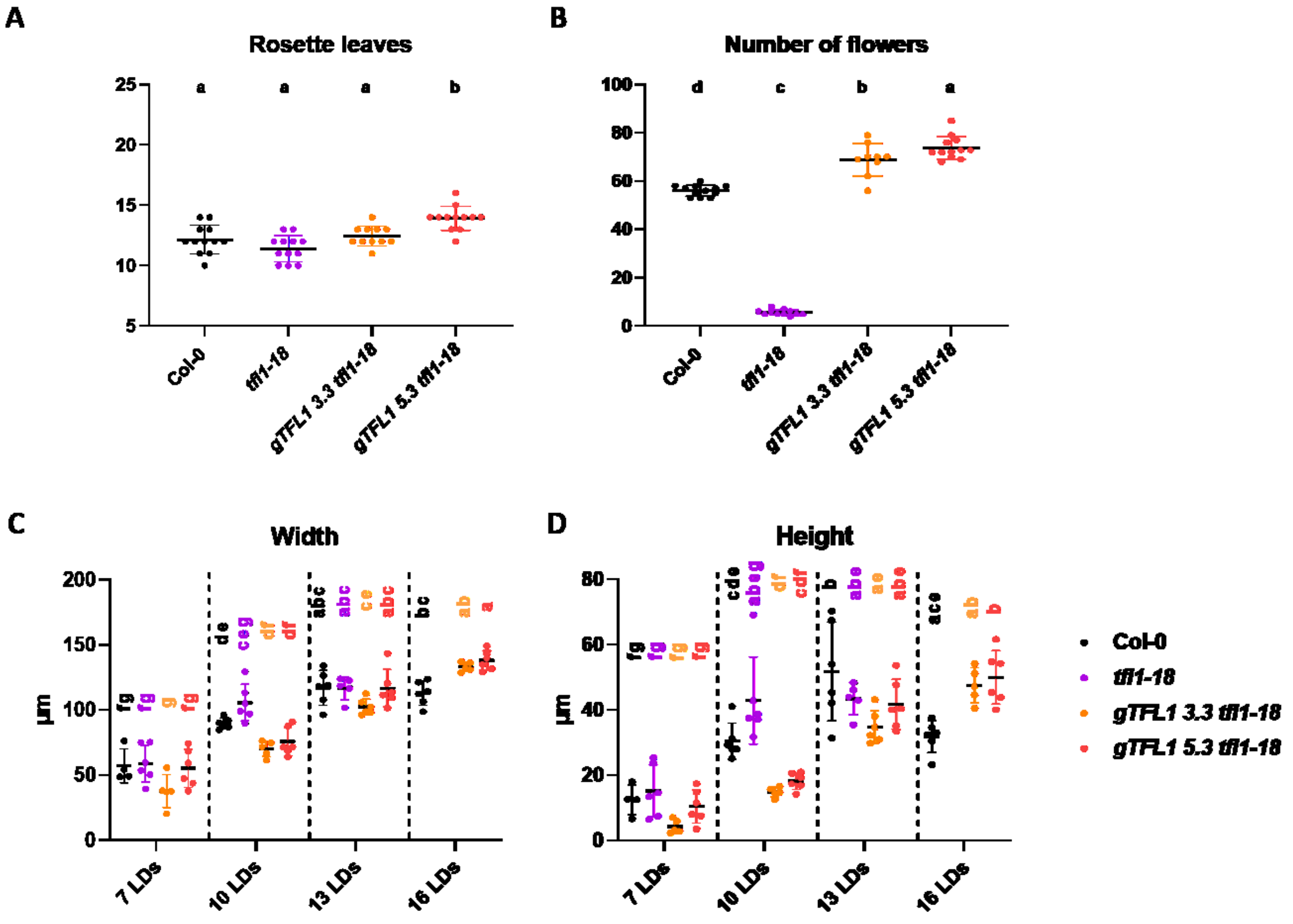

Fig. S3. *gTFL1* lines complement *tfl1-18* early flowering and prevent terminal flower formation.

- **A)** Complementation assay for the marker lines *gTFL1:VENUS tfl1-18* line 3.3 and 5.3 compared with Col-0 and *tfl1-18* in terms of number of rosette leaves.
- **B)** Complementation assay for the marker lines *gTFL1:VENUS tfl1-18* line 3.3 and 5.3 compared with Col-0 and *tfl1-18* in terms of number of flowers.
- **C)** Complementation assay for the marker lines *gTFL1:VENUS tfl1-18* line 3.3 and 5.3 compared with Col-0 and *tfl1-18* in terms of meristem width during floral transition.
- **D)** Complementation assay for the marker lines *gTFL1:VENUS tfl1-18* line 3.3 and 5.3 compared with Col-0 and *tfl1-18* in terms of meristem height during floral transition.

Development • Supplementary information

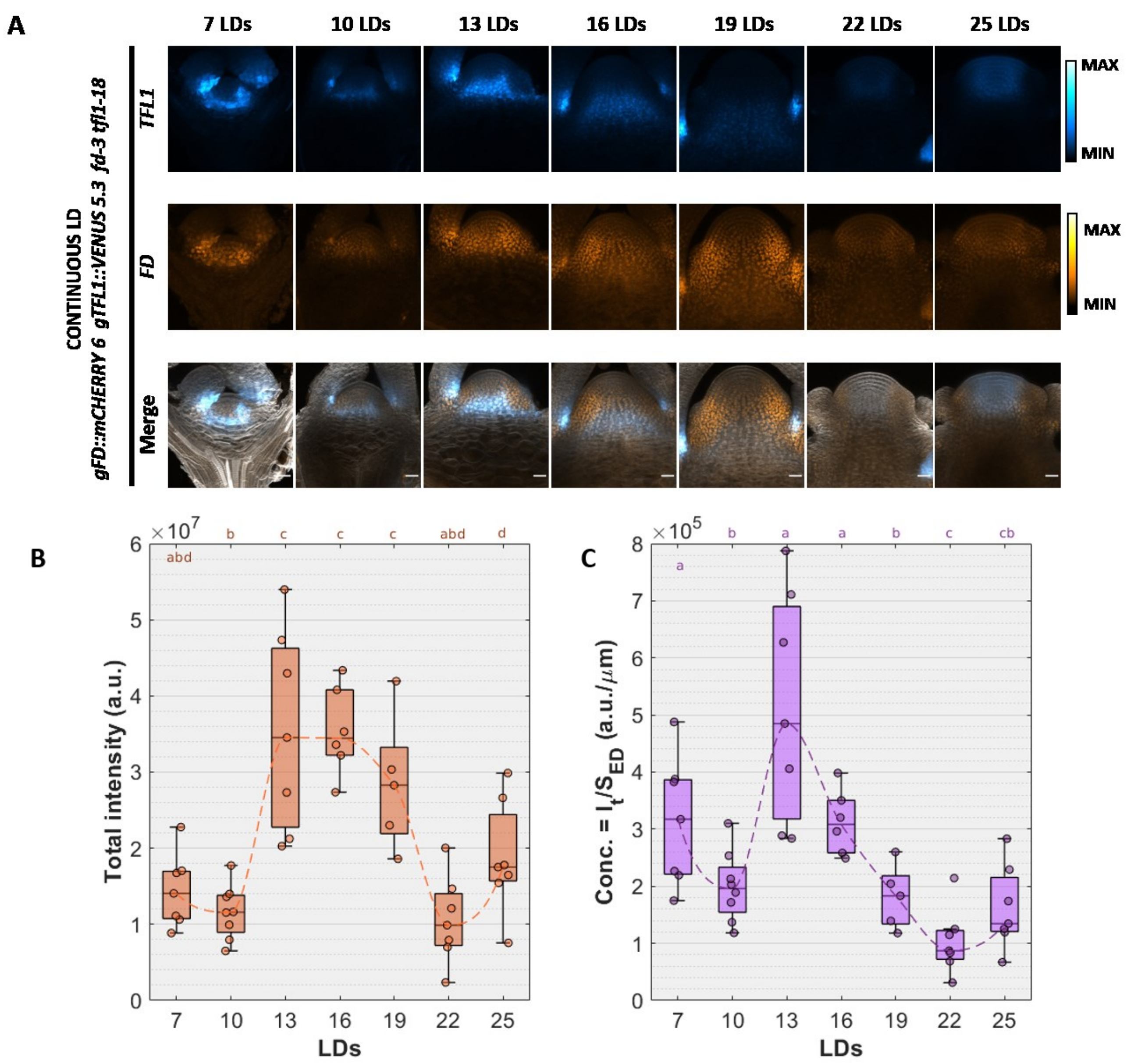

Fig. S4. Quantification of TFL1 intensity profile during floral transition.

- **A)** TFL1-VENUS (in cyan hot) and FD-mCHERRY (in orange hot) localization in the shoot apical meristem under long-day (LD) conditions. *gTFL1:VENUS* line 5.3 and *gFD:mCHERRY* line 6 were used. This image shows the entire time course; these images are partially reproduced in Fig. 2A.
- **B)** Box plots showing total intensity (I_t) measured in grey value (arbitrary unit: a.u.) of TFL1-VENUS signal within the defined expression domain at each time point during floral transition (Suppl. Methods 1). Dots represent individual samples. The dashed line represents the median trend with a cubic interpolation. Median values were compared using the Wilcoxon rank sum test (α=0.05).
- **C)** Box plots displaying concentration of TFL1-VENUS protein within the expression domain during floral transition for each time point. The concentration was measured as the ratio between the total intensity (I_t) and the size of the expression domain (S_{ED}) (grey value/micrometres). Dots represent individual samples. The dashed line represents the median trend with a cubic interpolation. Median values were compared using the Wilcoxon rank sum test (α=0.05).

Development • Supplementary information

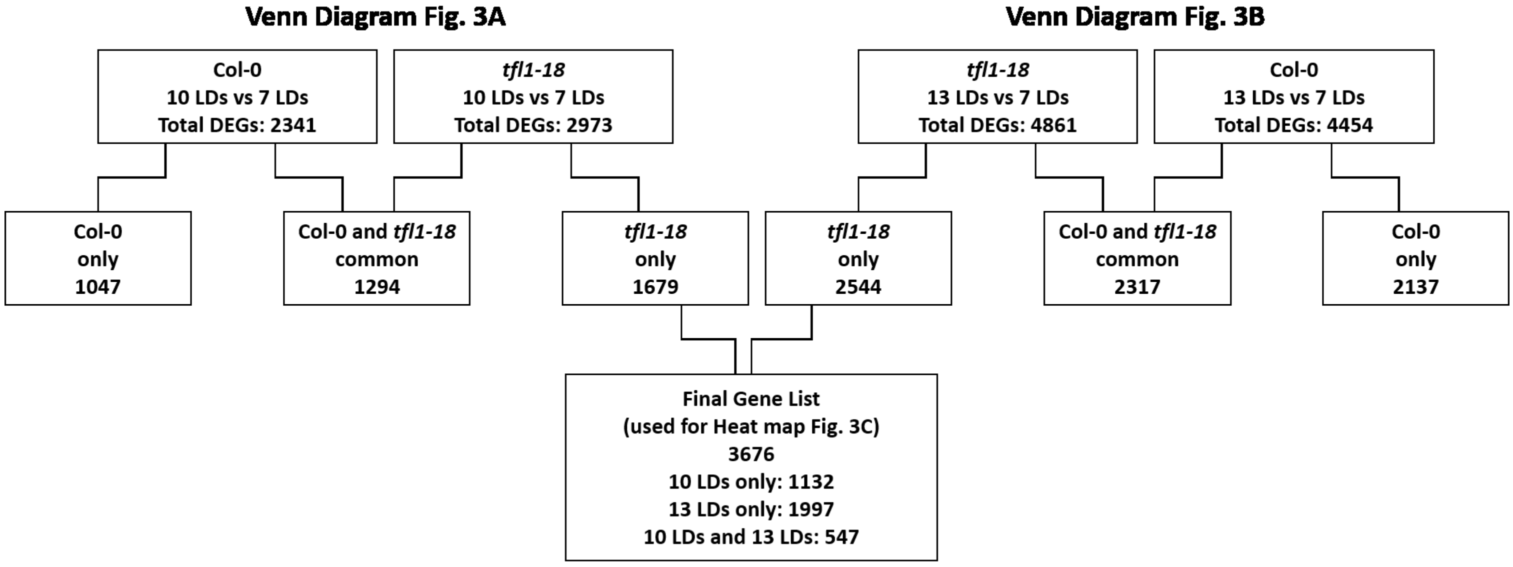

Fig. S5. Schematic representation of the comparison done for the RNA-sequencing analysis

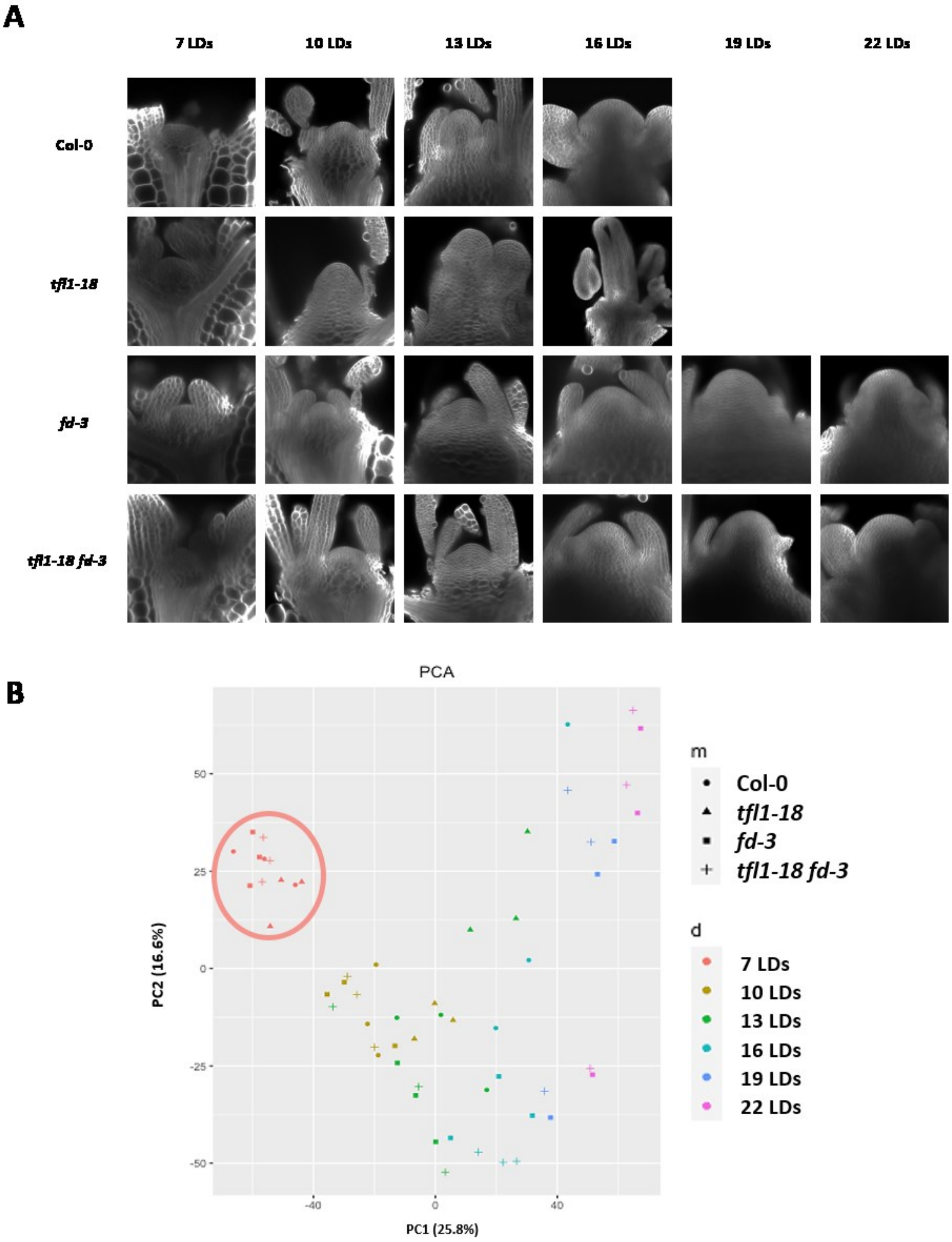

Fig. S6. Morphology of meristems at the time used for the RNA-sequencing.

A) Confocal images of meristems of Col-0, *tfl1-18*, *fd-3* and *tfl1-18 fd-3* during floral transition. The samples were imaged at the same time points as the RNA-seq samples.

B) Principal component analysis (PCA) matrix for all samples used for RNA-seq. At 7 LDs, the number of differentially expressed genes were 48, 164, 322 for the comparisons of Col-0 vs. *tfl1-18*, *fd-3* and *tfl1-18 fd-3*, respectively.

Development • Supplementary information

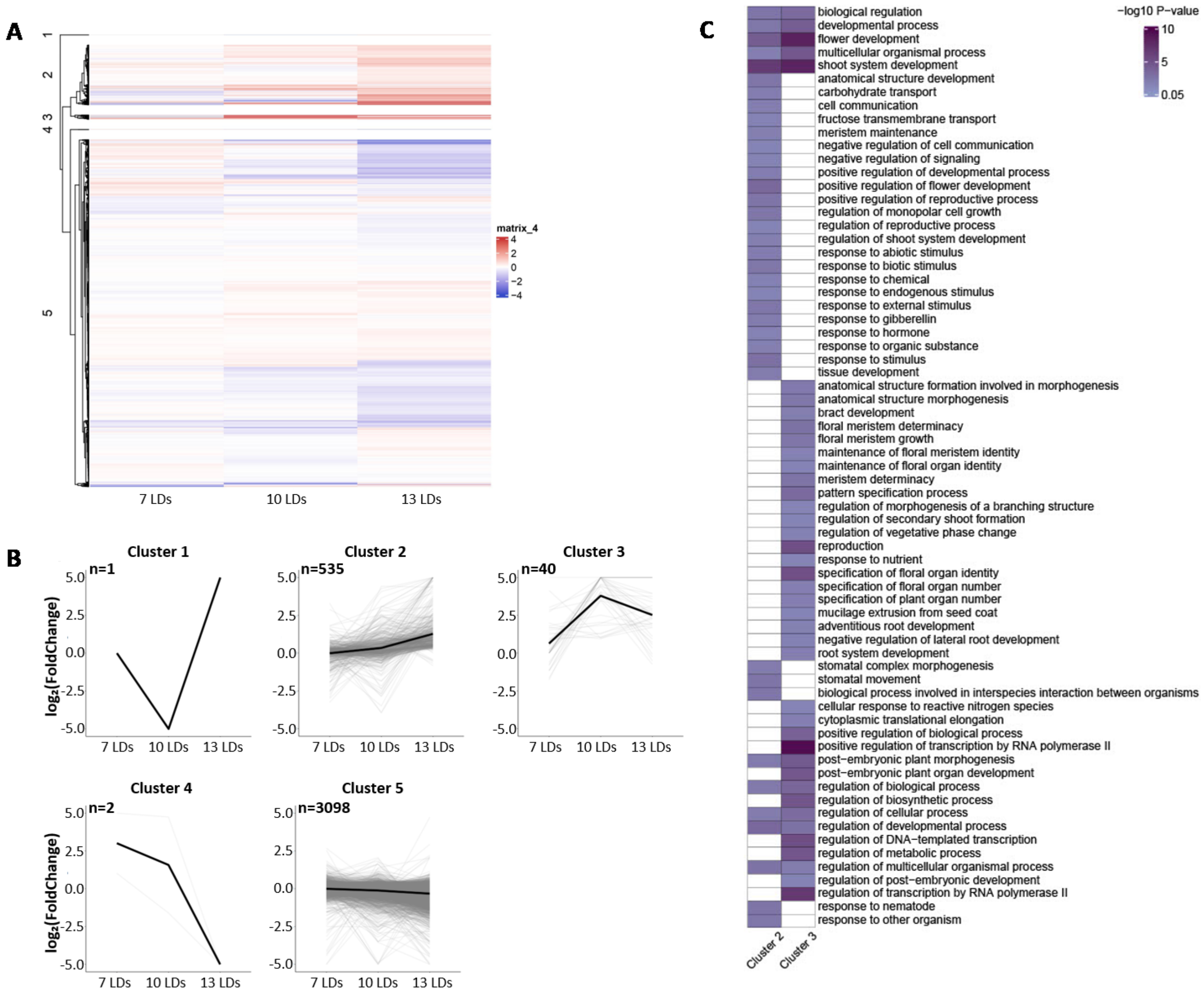

Fig. S7. Cluster analysis and GO enrichment of time resolved *tfl1-18* RNA-sequencing

- **A)** Heat map showing fold change in gene expression between *tfl1-18* and Col-0 for genes in all clusters at 7, 10 and 13 LDs.
- **B)** Representation of gene clusters from the classification in (A). The mean fold change for each cluster is shown as a bold line; n represents the number of genes in each cluster.
- **C)** GO enrichment terms for Clusters 2 and 3.

Development • Supplementary information

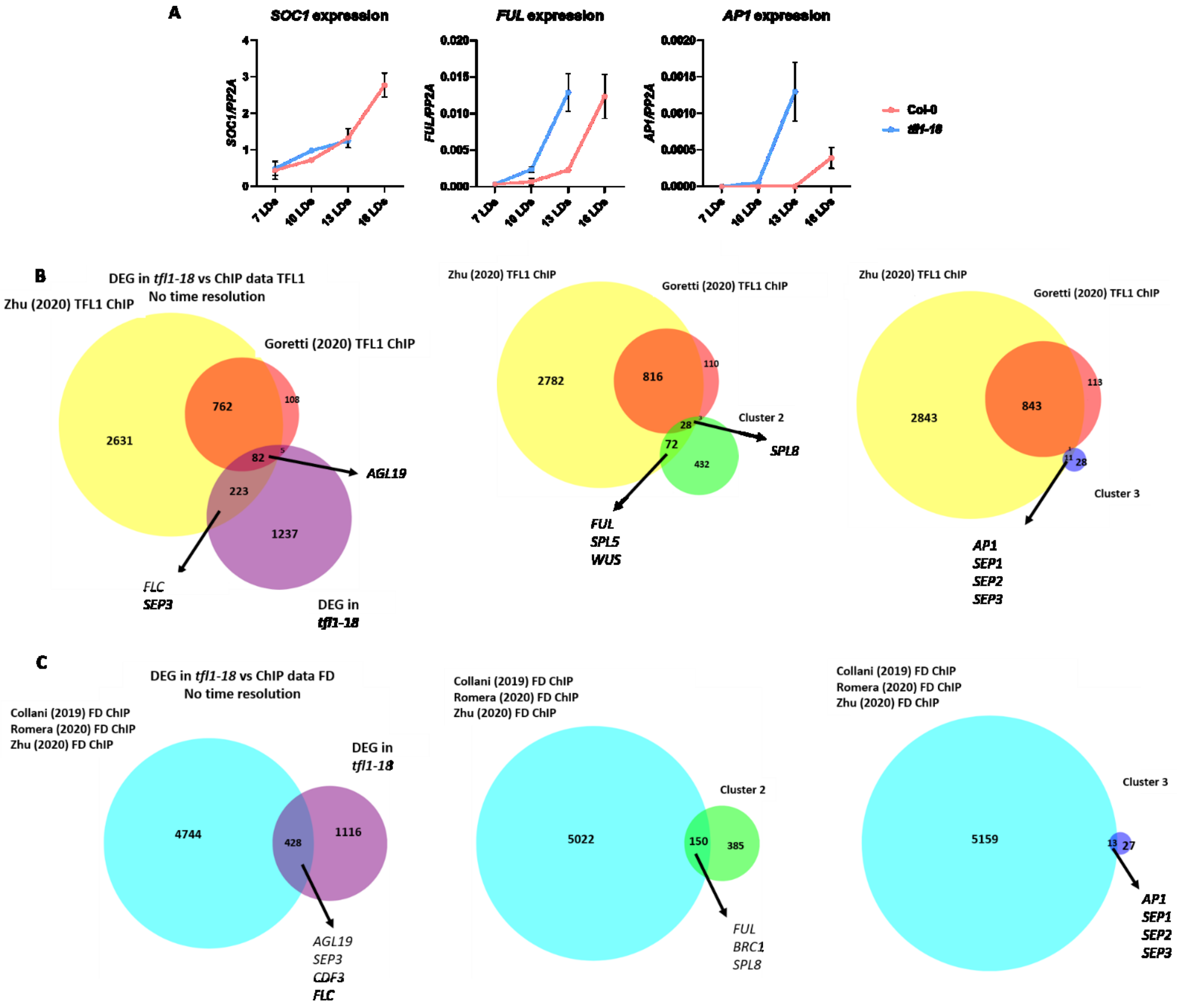

Fig. S8. Comparison between available ChIP-seq data and *tfl1-18* RNA-seq DEGs.

A) Expression of *SOC1, FUL* and *AP1* on enriched meristems of Col-0 and *tfl1-18* during floral transition. The graph represents RT-PCR data ± SD normalised against the expression of *PROTEIN PHOSPHATASE 2A* (*PP2A*) for two biological replicates.

B) Comparison of available TFL1 ChIP datasets with all the differentially expressed genes from Clusters 2 and 3 during floral transition in *tfl1-18*. Fisher´s exact test was performed to verify the relevance of the intersection among differentially expressed genes between *tfl1-18* and ChIP data. The *p*-values were 1.21E-15, 2.89E-05 and 0.003900459, respectively.

C) Comparison of available FD ChIP datasets with all the differentially expressed genes from Clusters 2 and 3 during floral transition in *tfl1-18*. Fisher´s exact test was performed to verify the relevance of intersection among differentially expressed genes between *tfl1-18* and ChIP data. The *p*-values were 1.45E-23, 2.13E-09 and 0.017548233, respectively.

Development • Supplementary information

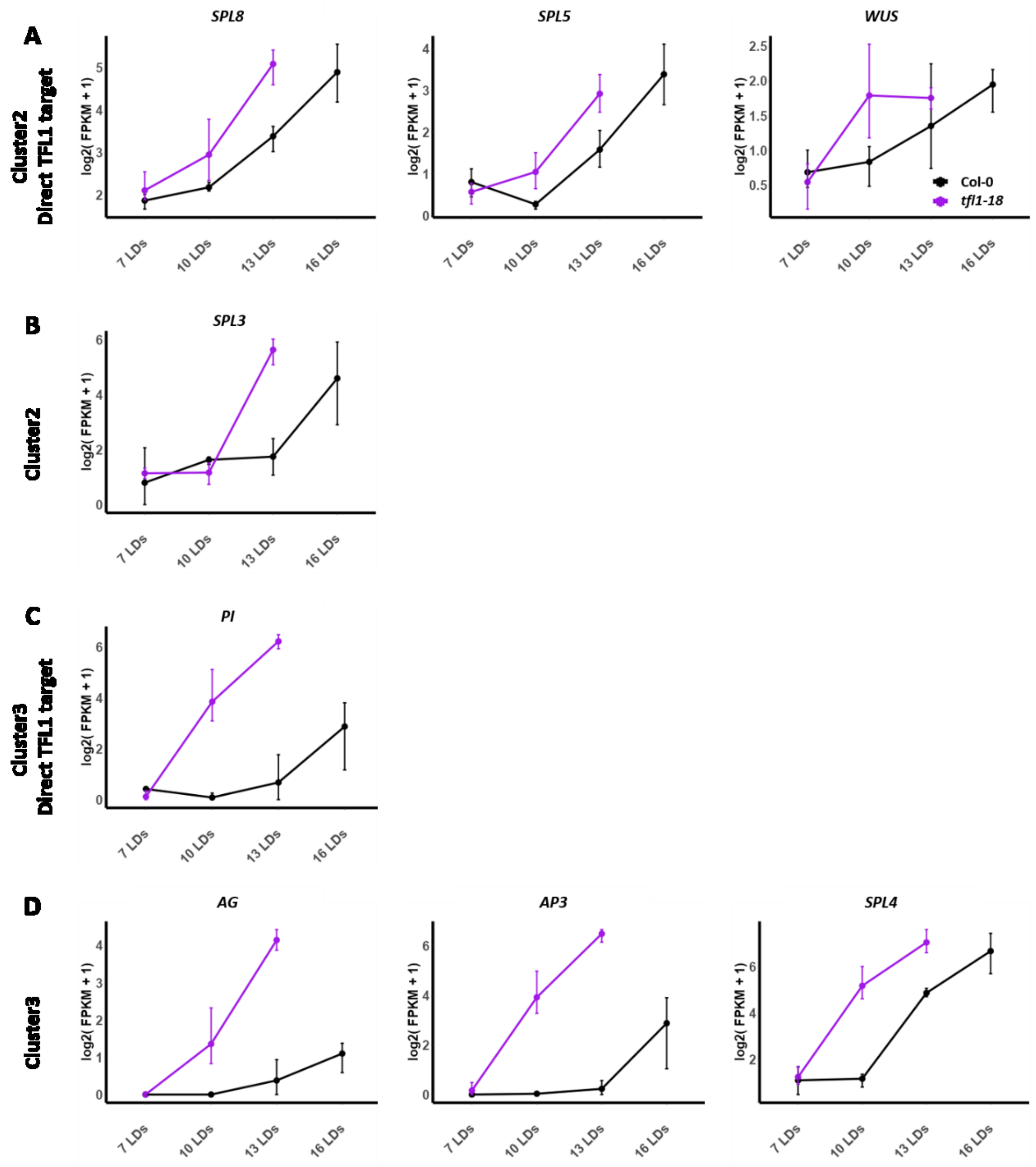

Fig. S9. Example of genes of Cluster 2 and Cluster 3.

A) SPL8, SPL5 and WUS are genes of Cluster 2 and they are also directly bound by TFL1 in published ChIP-sequencing.
B) SPL3 is a gene of Cluster 2, but it is not directly bound by TFL1 in published ChIP-sequencing.
C) PI is a gene of Cluster 3 and it is also directly bound by TFL1 in published ChIP-sequencing.
D) AG, AP3 and SPL4 are genes of Cluster 3, but they are not directly bound by TFL1 in published ChIP-sequencing.

Development • Supplementary information

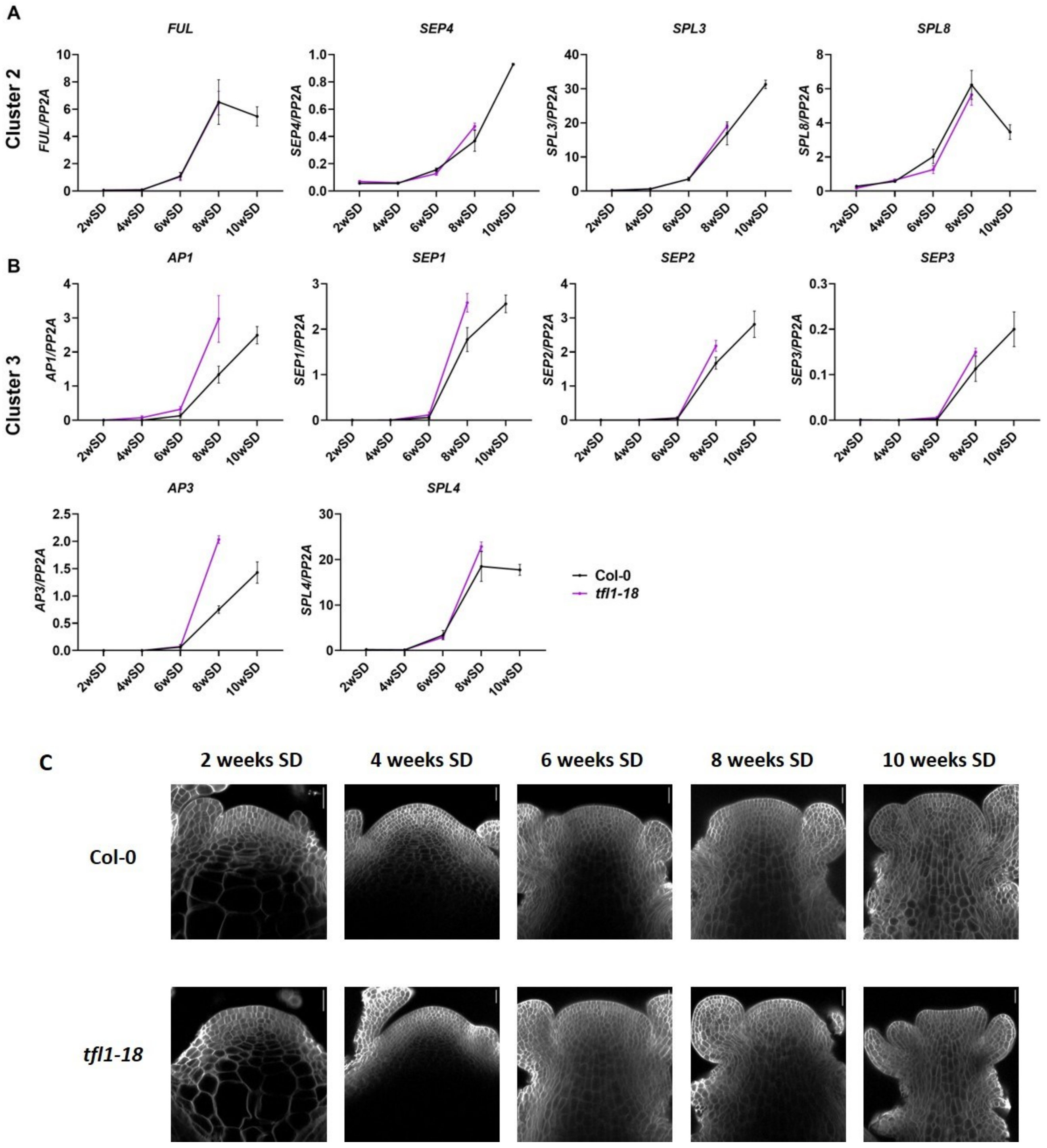

Fig. S10. Expression of Cluster 2 and Cluster 3 genes under SD conditions.

A) Expression profile of Cluster 2 genes *FUL*, *SEP4*, *SPL3* and *SPL8* under SDs in Col-0 and *tfl1-18*.

B) Expression profile of Cluster 3 genes *AP1*, *SEP1*, *SEP2*, *SEP3*, *AP3* and *SPL4* under SDs in Col-0 and *tfl1-18*.

C) Morphology of the meristems of Col-0 and *tfl1-18* under SDs time course. Terminal flower formation was detected in *tfl1-18* at 10 weeks SD.

Development • Supplementary information

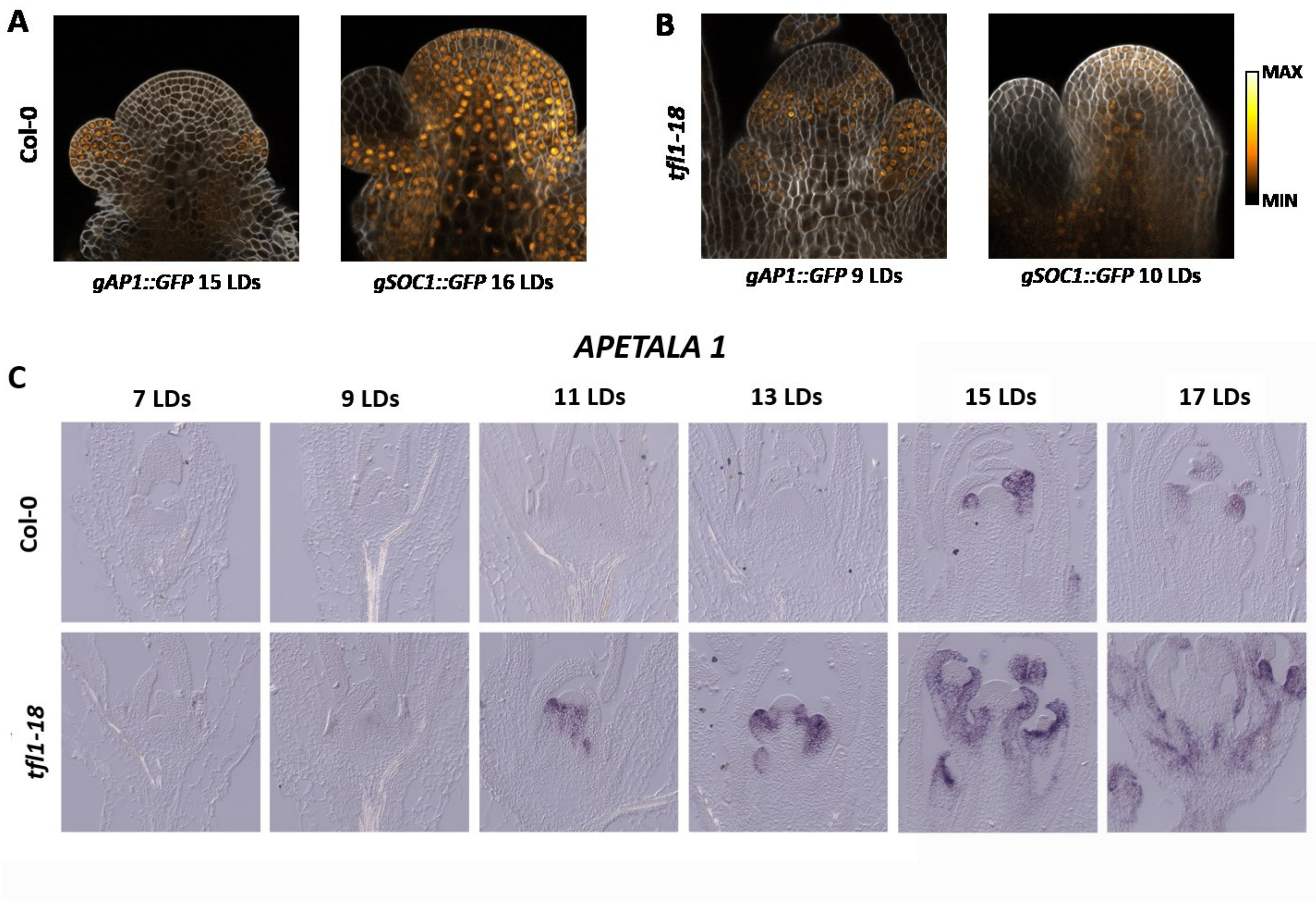

Fig. S11. AP1 and SOC1 show a complementary pathway in both Col-0 and *tfl1- 18.*

- **A)** AP1-GFP is present in floral primordia at the flanks of the inflorescence meristem in Col-0. SOC1-GFP expression is visible in the inflorescence meristem, but not in floral primordia in Col-0.
- **B)** AP1-GFP is present in the inflorescence meristem and in floral primordia of *tfl1-18*, whereas SOC1-GFP is visible in the inflorescence meristem, but not in floral primordia of *tfl1-18*.
- **C)** *In situ* hybridization of *AP1* in Col-0 and *tfl1-18* during floral transition.

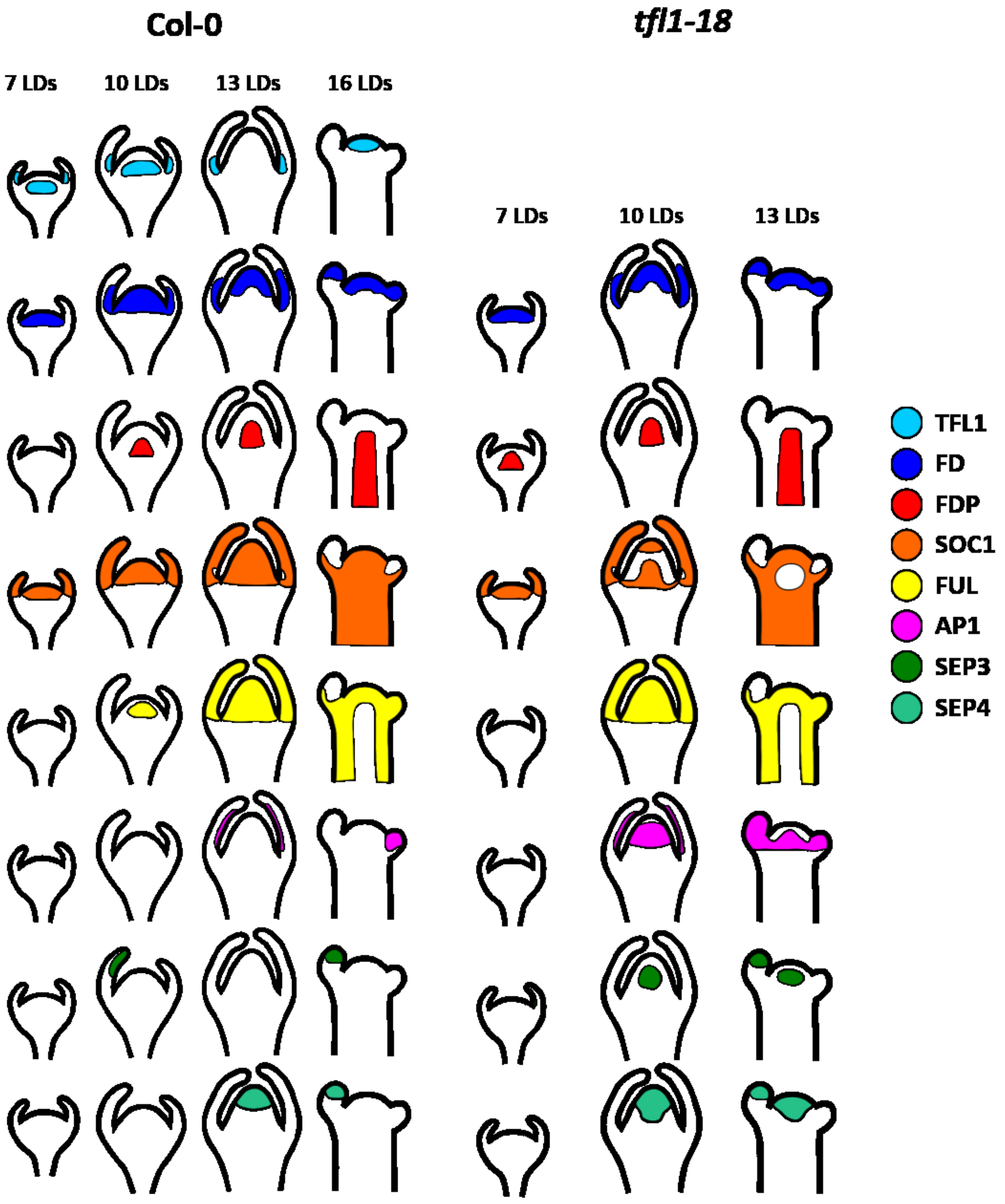

Fig. S12. Schematic expression of different genes involved in floral transition and flower development in Col-0 and *tfl1-18*

Schematic representation of the expression domains of TFL1, FD, FDP, SOC1, FUL, AP1, SEP3 and *SEP4* in Col-0 and *tfl1-18* during floral transition. *TFL1*, *FD*, *FDP*, *SOC1*, *FUL*, *AP1* and *SEP3* scheme are based on confocal acquisition and protein distribution, *SEP4* scheme is based on *in situ* hybridization and mRNA expression.

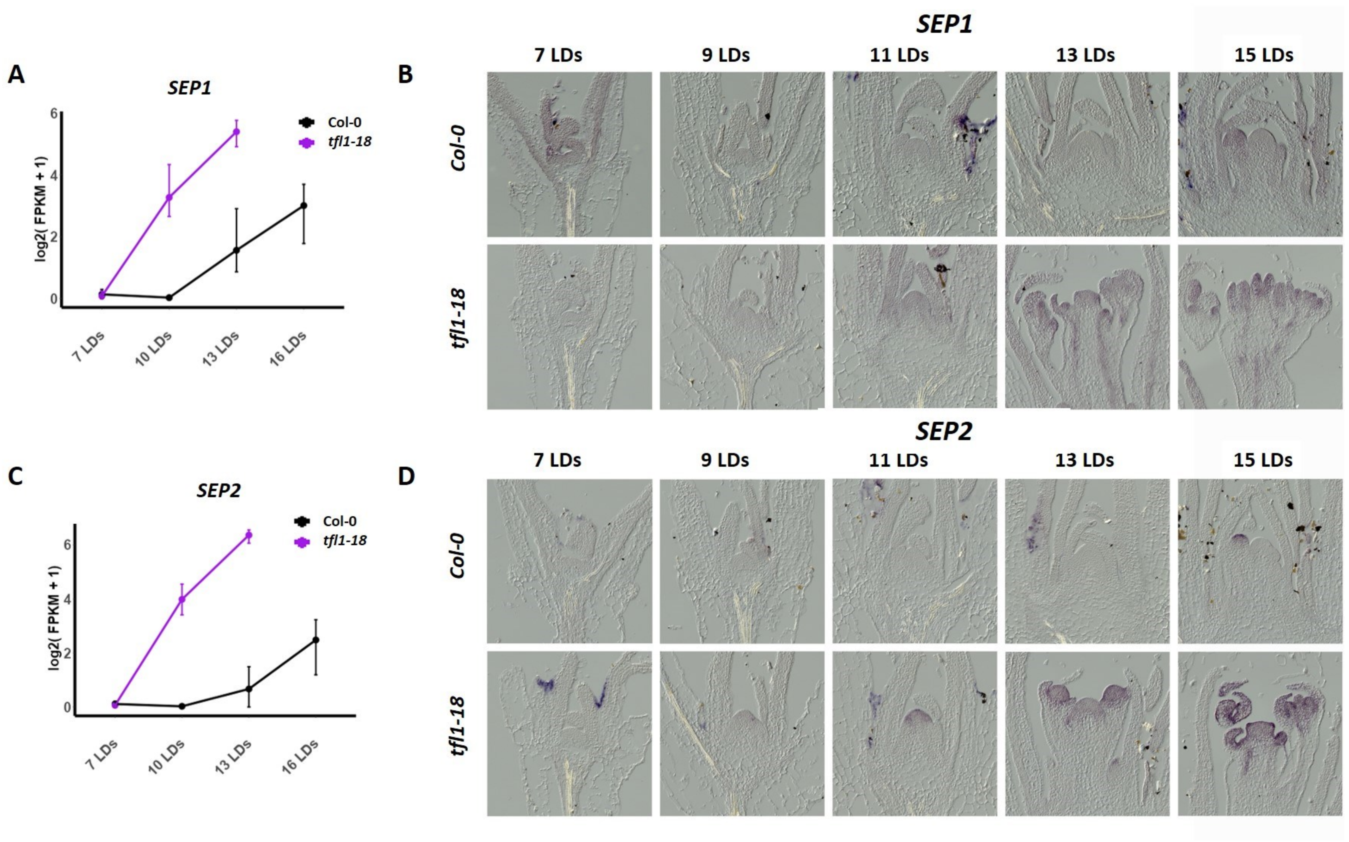

Fig. S13. Expression and *in situ* hybridization of *SEP1* and *SEP2* in Col-0 and *tfl1- 18* during floral transition

- **A)** *SEP1* expression in Col-0 and *tfl1-18* during floral transition.
- **B)** *In situ* hybridization of *SEP1* in Col-0 and *tfl1-18* during floral transition.
- **C)** *SEP2* expression in Col-0 and *tfl1-18* during floral transition.
- **D)** *In situ* hybridization of *SEP2* in Col-0 and *tfl1-18* during floral transition.

Development • Supplementary information

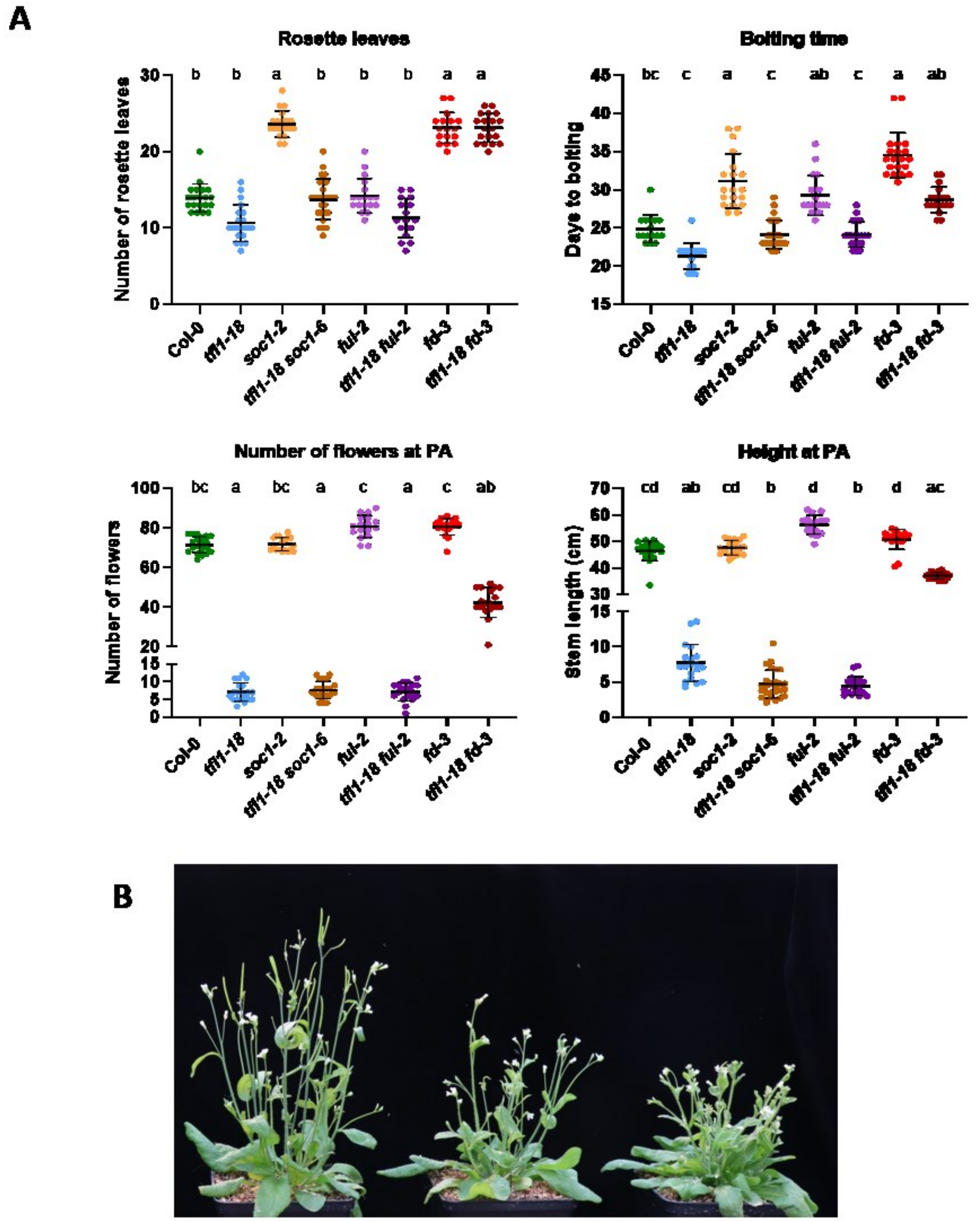

tfl1-18 *tfl1-18 ful-2* *tfl1-18 soc1-6*

Fig. S14. the double mutants *tfl1 ful* and *tfl1 soc1* do not complement *tfl1* phenotype

A) Phenotypic characterization of Col-0, *tfl1-18*, *soc1-2*, *tfl1-18 soc1-6*, *ful-2*, *tfl1-18 ful-2*, *fd-3*, *tfl1-18 fd-3*. The phenotypes analysed were the number of rosette leaves, days to bolting, number of flowers at proliferative arrest (PA), and total stem height at PA.

B) Photographs of *tfl1-18*, *tfl1-18 ful-2* and *tfl1-18 soc1-6* at PA.

Development • Supplementary information

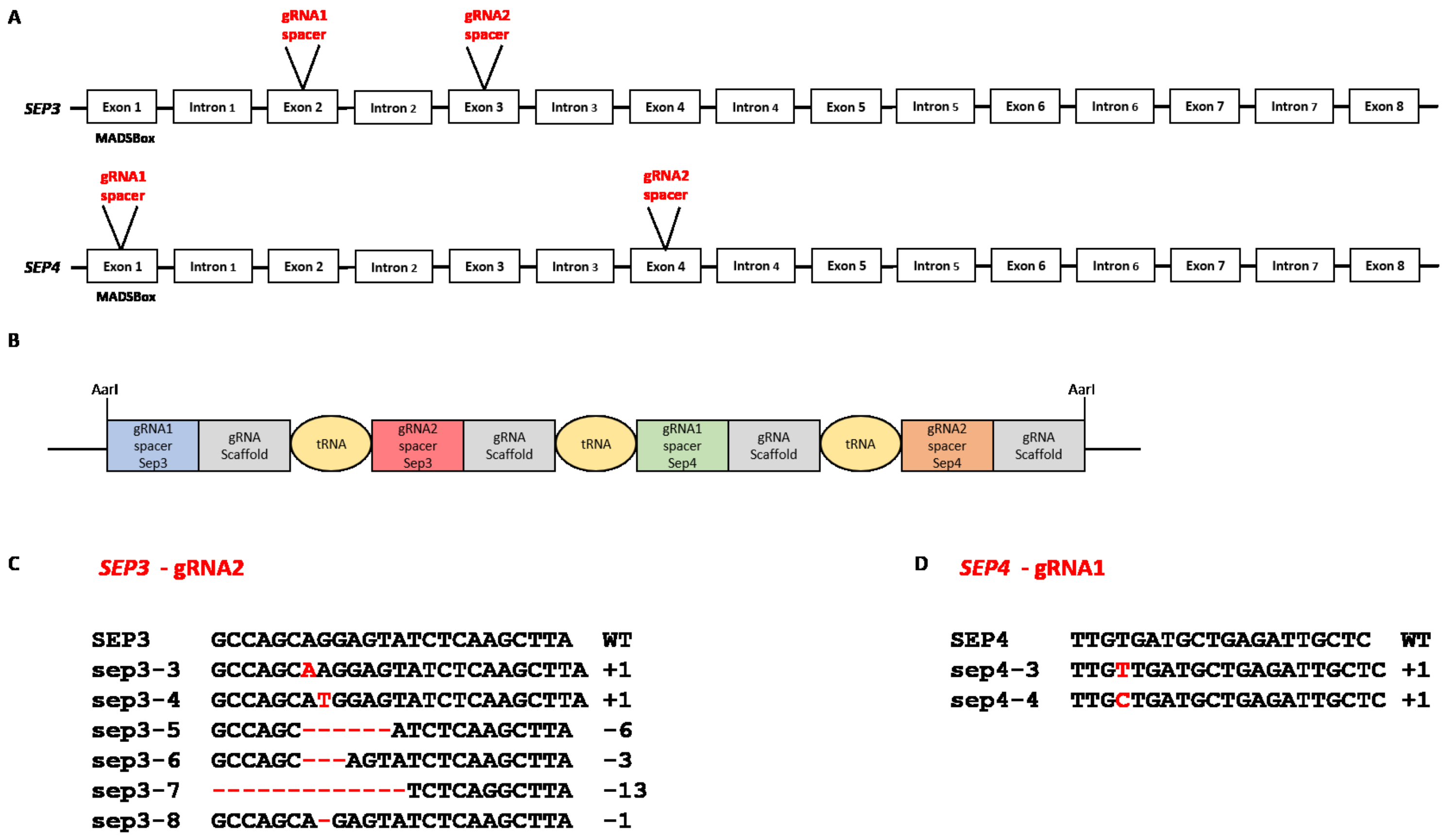

Fig. S15. New alleles obtained by CRISPR-Cas9 mutation of *SEP3* and *SEP4*.

- **A)** Scheme to show the guide design for CRISPR mutagenesis of *SEP3* and *SEP4*. Two guide RNAs were designed for both genes.
- **B)** Schematic diagram showing the cloning strategy of the four guide RNAs.
- **C)** Summary of the alleles generated by CRISPR mutation.

Development • Supplementary information

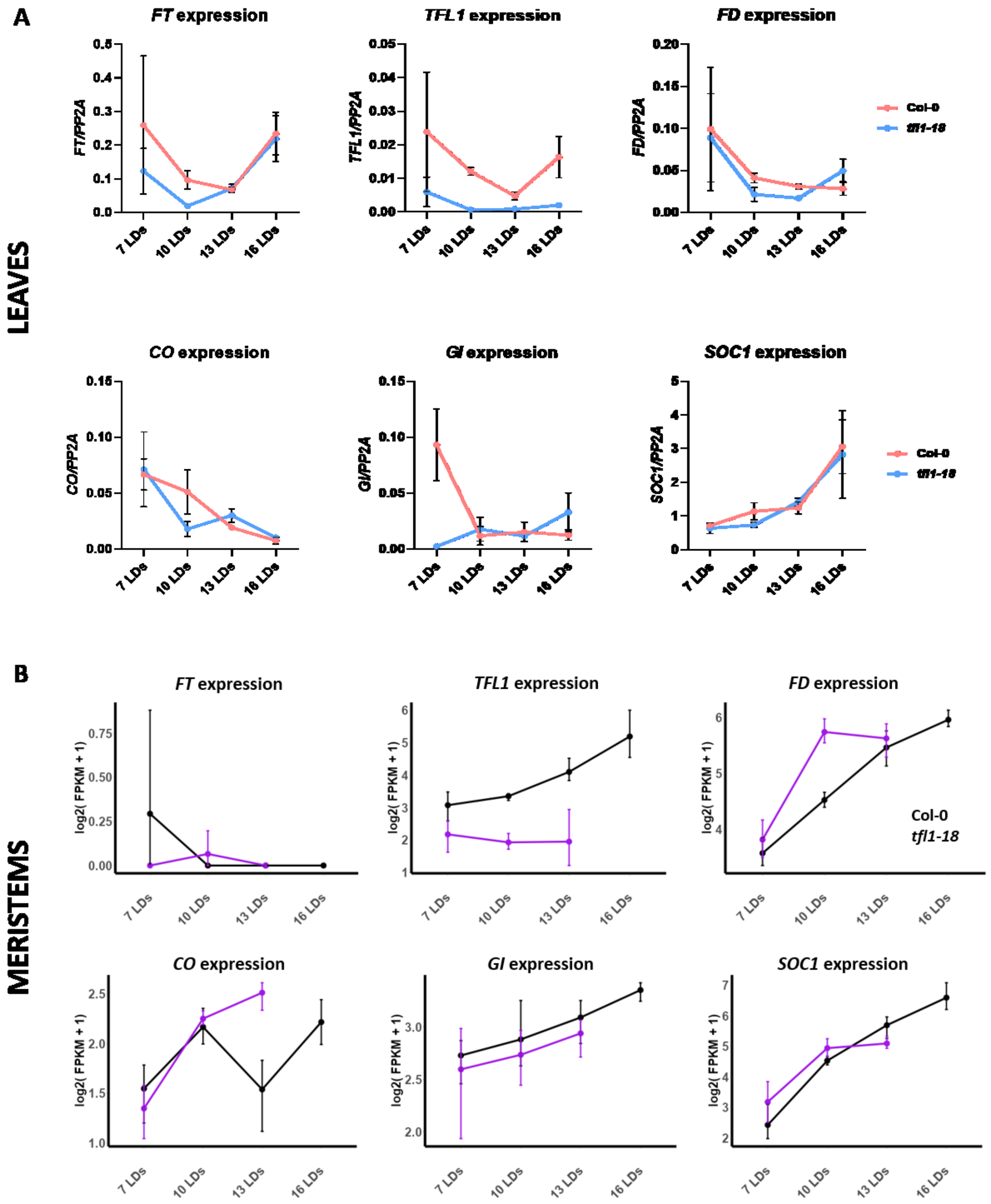

Fig. S16. The expression in leaves of genes related to photoperiod perception is not affected in *tfl1-18.*

A) Levels of *FT*, *TFL1*, *FD*, *CO*, *GI* and *SOC1* mRNAs in Col-0 and *tfl1-18* leaves harvested at ZT 7–16. The graphs represent RT-PCR data ± SD normalised against the expression of *PROTEIN PHOSPHATASE 2A* (*PP2A*) for three biological replicates.

B) Levels of *FT*, *TFL1*, *FD*, *CO*, *GI* and *SOC1* mRNAs in apices of Col-0 and *tfl1-18* as extracted from the RNA-seq data.

Development • Supplementary information

Supplementary Materials and Methods

Confocal fluorescence z-stacks were processed and analyzed using Matlab custom made code (https://gitlab.com/slcu/teamHJ/pau/RegionsAnalysis/-/releases/TFL1_FD_code). The main goal of this analysis was to extract reproducible measures of fluorescence intensity within the SAM. To do that, a semi-automatic pipeline was developed, which is described as follows.

As a start, a pre-processing step was needed; due to the difference in the resolution between the xy plane and the z-direction (depth), the z-stack was resized by increasing the number of slices in the z-direction through a bicubic interpolation[1] to obtain an homogeneous volumetric resolution. Fluorescence signal was also detected in SAM biological boundaries.

After this pre-processing step, a 3D paraboloid mask was constructed using the curvature of the meristem (Suppl. Fig. S17ED) in order to exclude fluorescence signals outside the region of interest and quantify only the fluorescence intensity within the meristematic region. To do that, a stack-slice interval that contains the apex of the meristem was selected and the cell wall signal present within this interval was projected in each orthogonal planes (xy and yz) (see Suppl. Fig. S17A and B). Then, two curved lines following the parabolic SAM's outline were drawn in the sum of slice projections of the xy and yz orthogonal planes (Suppl. Fig. S17B). Afterwards, a parabolic fitting of the two drawn lines was performed (Suppl. Fig. S17C). From the two orthogonal parabolas fitted per each z-stack, the apex was computed to derive the equation for the 3D paraboloid. The z_o coordinate of the paraboloid was determined from averaging the apices of the orthogonal parabolas (see Suppl. Fig. S17D). The parameter *a* in the parabola equation was used to substitute the denominator terms in the paraboloid equation (c_1^2 and c_2^2) (Suppl. Fig. S17D). By doing this, it is ensured the paraboloid matches the linear and quadratic terms of each of the parabola's equations at $y=y_o$ and $x=x_o$ respectively (turquoise and purple parabolas in Suppl. Fig. S17D). Then, a 2D parabolic mask was defined in terms of the image pixel position for each of the z-stack slices based on the paraboloid previously constructed (Suppl. Fig. S17E).

Each slice of the z-stack was multiplied times the corresponding 2D parabolic mask that sets to zero all intensity values of the pixels outside the paraboloid (pixels above red parabola in Suppl. Fig. S17E). The sum of the slice projection (xy and yz planes) was then computed (Suppl. Fig. S17F). A vertical line was marked out in this projection starting at the apex coordinates reaching the bottom of the image (red line in Suppl. Fig. S17F) -. The width of this line can be automatically selected (1 µm width, Suppl. Fig. S17F). Finally, the intensity profile along this line was extracted to be later analyzed (Suppl. Fig. S17G).

In some cases, stronger fluorescence signal appeared specifically at boundary regions of new primordia, which did not want to take into account in our quantification. When using paraboloid masks constructed using the same curvature derived from the parabolas, such signals were biasing the quantification. In order to exclude the fluorescence signal at such meristematic biological boundaries, the paraboloid curvature was increased respect to the original such as $a' = a/\alpha$ being a' the curvature of the new paraboloid and α<1µm the image resolution (Suppl. Fig. S17D).

---

1 The resolutions in the xy-plane are identical for all images, except those taken at 7LDs (vegetative meristem).

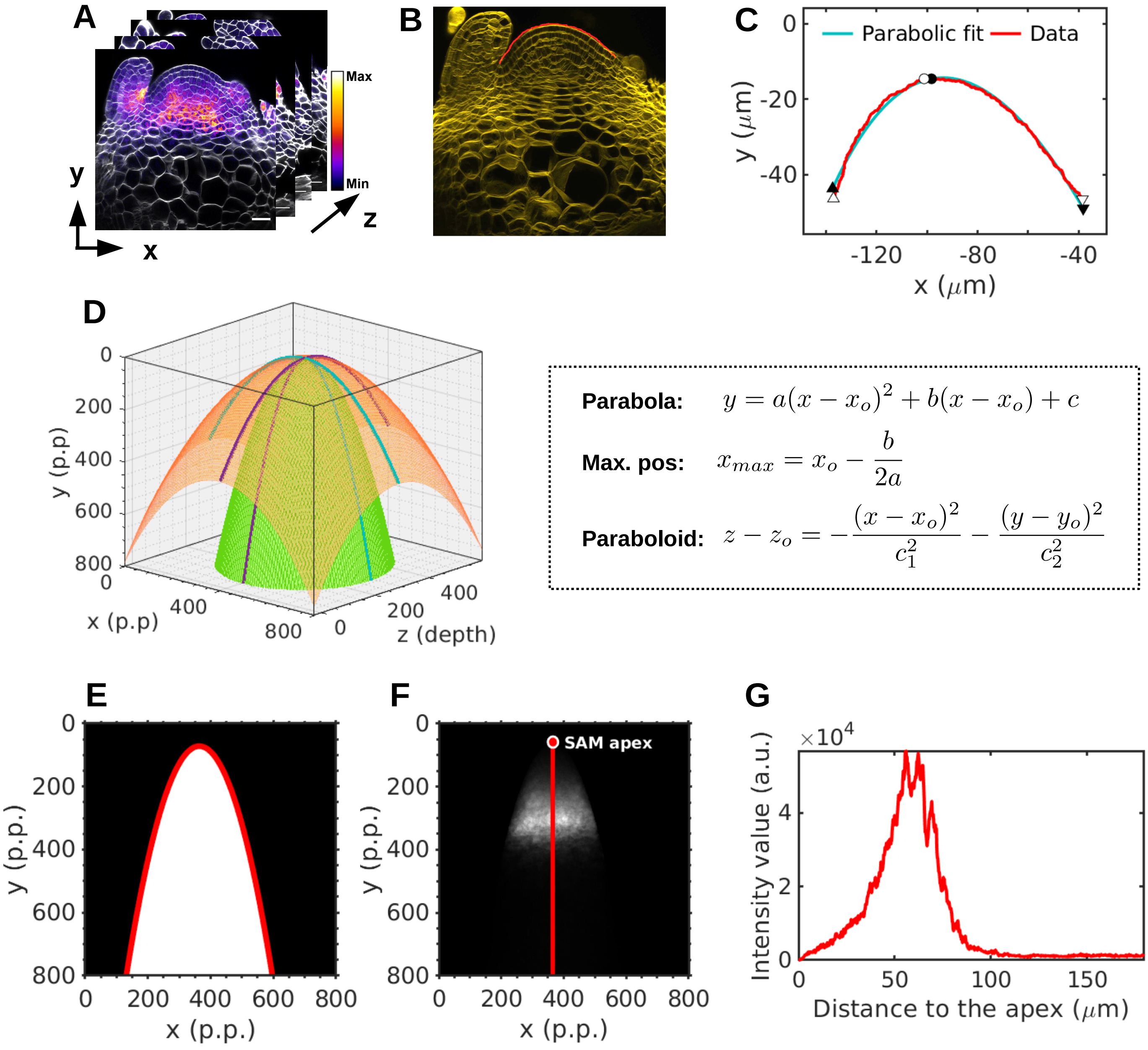

Fig. S17. Fluorescence quantification explanation pipeline. (A) SAM fluorescence confocal microscopy images were obtained from the lateral side. In the image: slices from z-stack meristem grown at LDs conditions at 13 days after sowing (DAS). Membrane marker channel (in white) and TFL1-fluorescence signal (plant with genotype *gFD::mCherry 6 gTFL1::Venus 5.3 FD-3 tfl1-18,* in fire-color look up table from Fiji). White bar indicates 20 µm. The acquisition parameters are described in *Materials and Methods.* (B) A curved line (in red) follows the SAM outline until the beginning of the primordia on the sum of slice projection of certain z-stack slice interval. (C) Parabolic fitting of the previous parabola. Triangles mark the beginning and end points of the drawn curve line (white) and fitted parabola (black). Circles represent the apex position for drawn curve line (white) and fitted parabola (black). (D) 3D paraboloid masks built from the previously extracted orthogonal parabolas. In orange, paraboloid using original curvature values. In green, paraboloid with higher curvature to exclude from the quantification the fluorescence signal in new emerging primordia boundaries. On the right, main set of equations describing the 2D parabola in the xy-plane, its maximum position and the 3D paraboloid. The 2D equation of the parabola in the zy-plane is omitted for simplicity. (E) 2D parabolic mask from the 3D paraboloid as a function of image pixel position (xy-plane). When applied to the corresponding z-stack slice, pixels whose positions lie above the red curve will have their intensity values set to zero. (F) Sum of the slice projection (xy-plane) after the application of the 3D paraboloid mask. A red vertical line is marked from the SAM apex coordinates to the lowermost part of the image. (G) Intensity profile as a function of apex distance (in µm) extracted from the previous red vertical line.

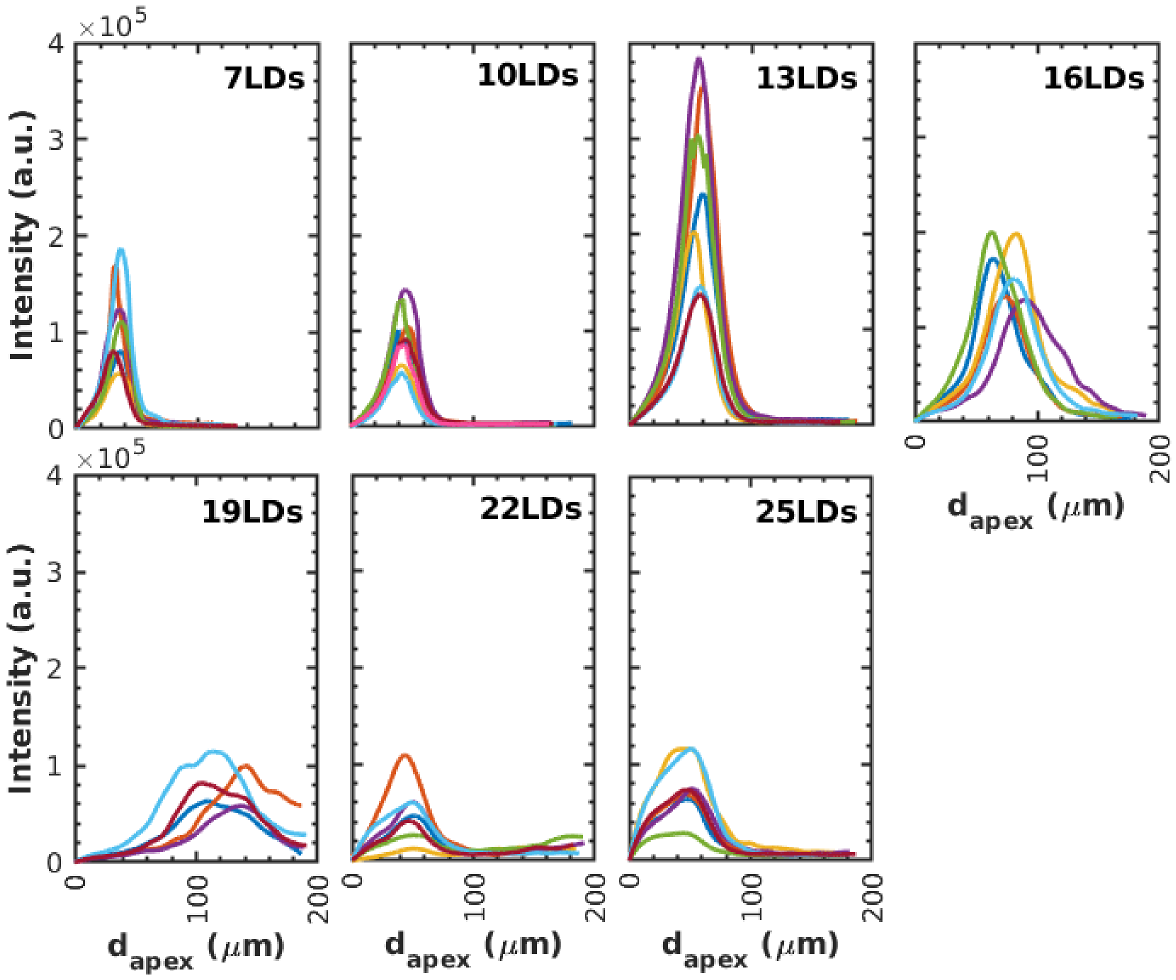

Fig. S18. Intensity profiles of TFL1-Venus along the longitudinal section of the meristem as a function of apex distance (µm) for different meristems (colors) at different temporal points (7-25 LDs). Intensity profiles were extracted from the xy sum-of-slice projection along a vertical line going from the apex of the meristem towards the bottom of the meristem. The intensity profiles shown were smoothened with a robust, quadratic filter in Matlab ("*rloess*" , window = 125 points). Number of samples by time point: 7, 8, 7, 6, 5, 7 and 7 for 7, 10, 13, 16, 19, 22 and 25 LDs respectively.

Development • Supplementary information

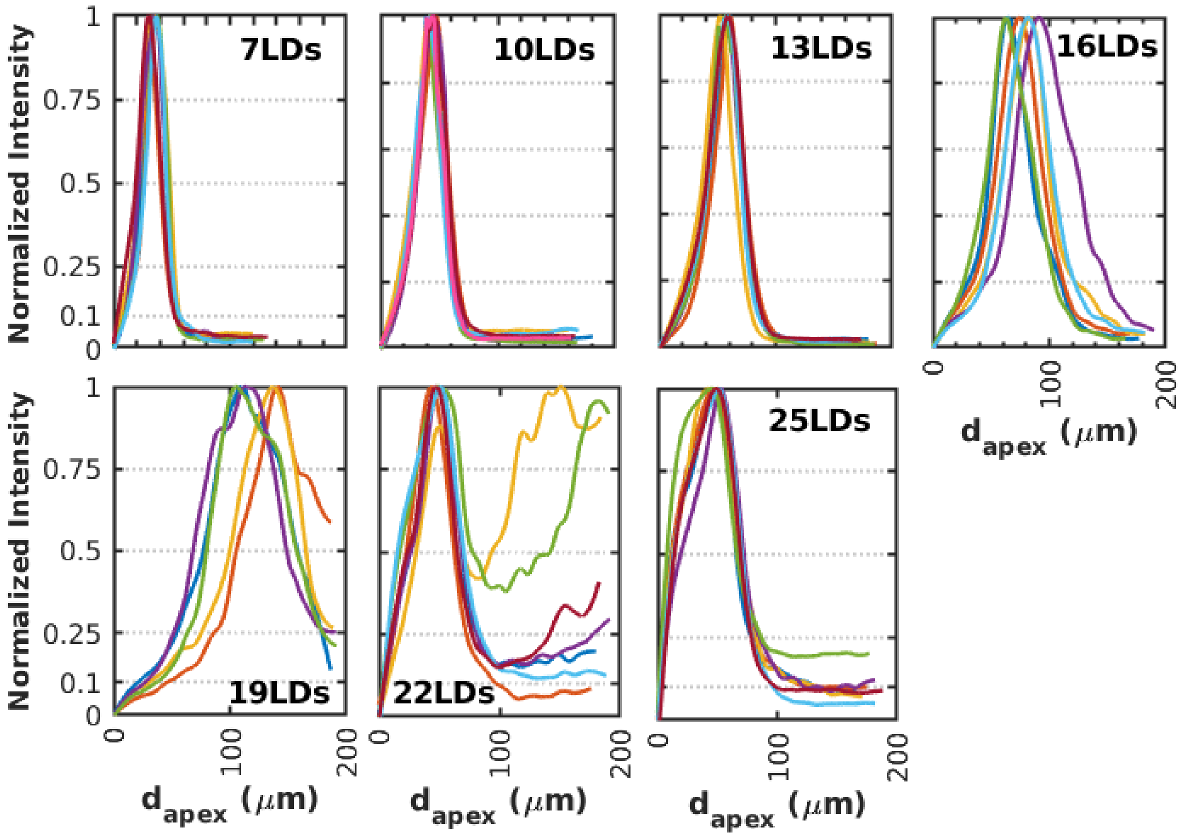

Fig. S19. Normalized intensity profiles extracted from the xy-sum-of-slice projection as a function of apex distance (μm) for different meristems (colors) at different temporal points (7-25 LDs) after the application of a smoothing (robust, quadratic) filter. For each intensity profile, each intensity data measurement is divided by the maximum intensity. The intersection point of the dashed gray lines with each profile corresponds to the initial and ending point of the expression domain for each threshold (0.10, 0.25, 0.5 and 0.75 of the maximum intensity). When this threshold is not reached – only with ending domain points, at the lower part of the SAM - , the point whose distance is minimum is taken instead. For the special case of the two bimodal intensity profiles at 22LDs (green and yellow lines), the algorithm focuses on the first peak when computing the maximum intensity reached in the profile.

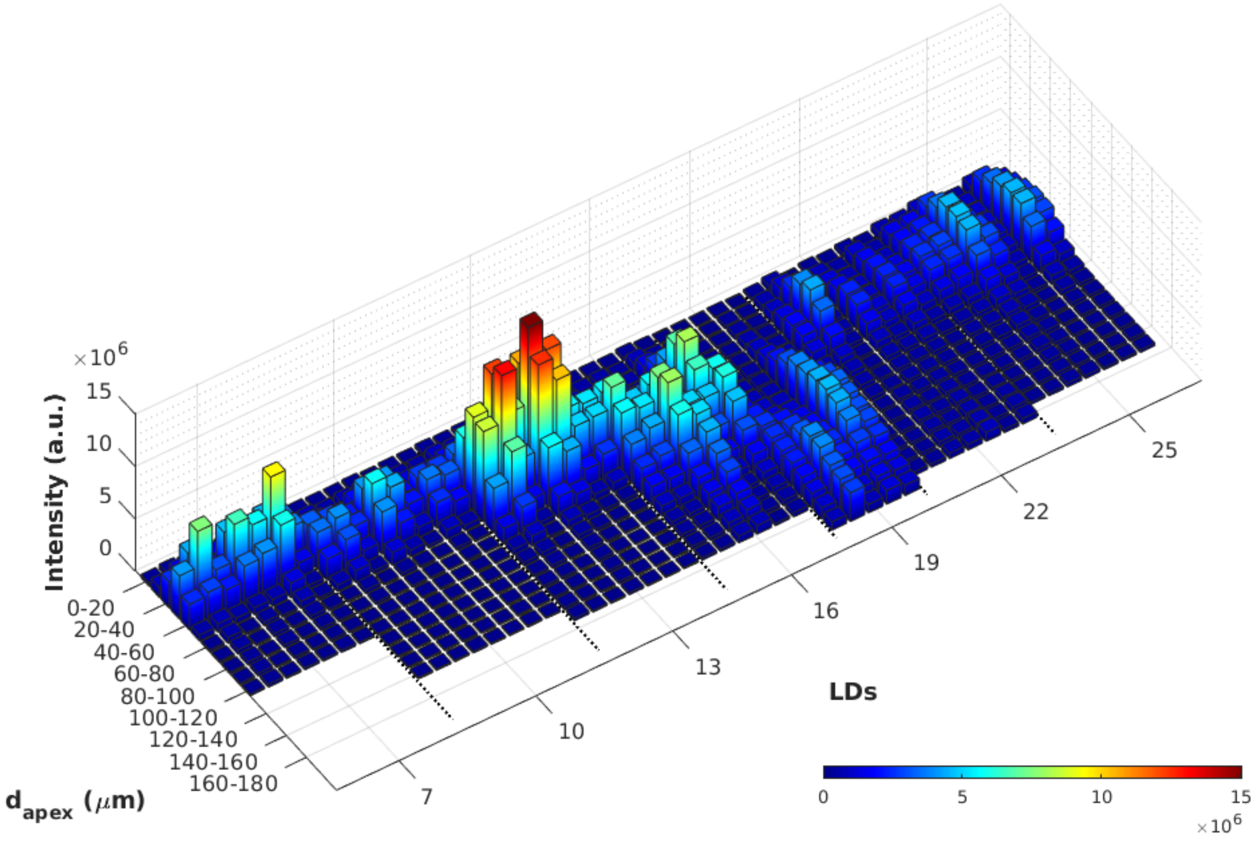

Fig. S20. TFL1-Venus total intensity per 10 μm-length interval as a function of the distance to the shoot apex at different time points. Each intensity profile was extracted from the xy-sum-of-slice projection. The color scale matches the height of each bar.

Development • Supplementary information

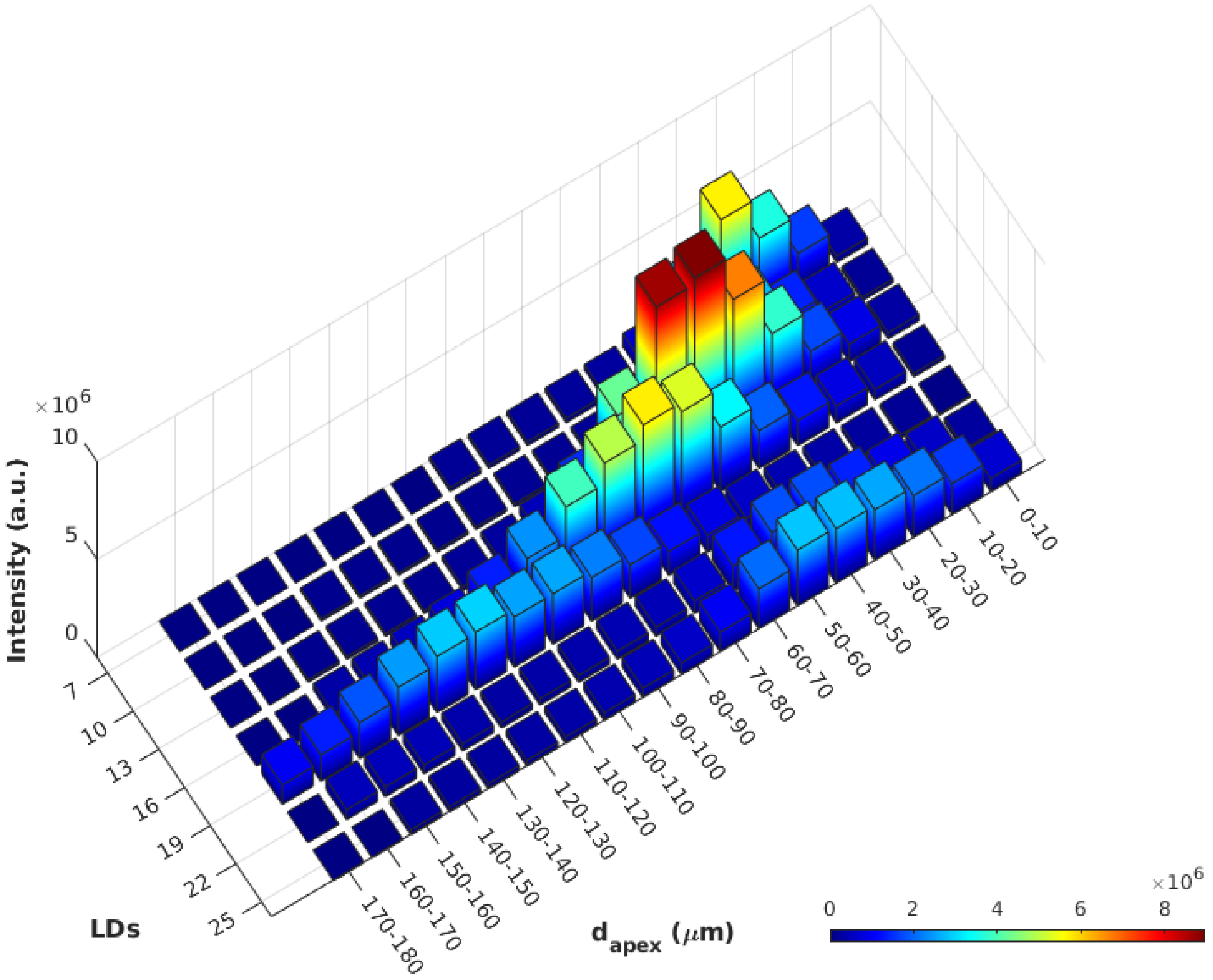

Fig. S21. TFL1-Venus median total intensity across the different repeats shown in Suppl. Fig. S20 per 10 µm-length interval as a function of the distance to the shoot apex at different time points. The color scale matches the height of each bar.

Development • Supplementary information

Table S1. RNA-sequencing analysis.

Available for download at
https://journals.biologists.com/dev/article-lookup/doi/10.1242/dev.202089#supplementary-data

Table S2. Primers sequences and PCR conditions.

Available for download at
https://journals.biologists.com/dev/article-lookup/doi/10.1242/dev.202089#supplementary-data

Table S3. Number of replicates for confocal acquisition.

Available for download at
https://journals.biologists.com/dev/article-lookup/doi/10.1242/dev.202089#supplementary-data

Table S4. RNA-sequencing data tables. This data table is available in Dryad repository in the "Fig.3 folder".

Available for download at
https://journals.biologists.com/dev/article-lookup/doi/10.1242/dev.202089#supplementary-data

Table S5

ini(ED) th.=0.1						
LDs	10	13	16	19	22	25
7	0.0420	0.0006	0.0012	0.0025	0.0239	0.0012
10		0.0006	0.0007	0.0016	0.0003	0.0003
13			**0.2494**	0.0051	0.0006	0.0006
16				0.0303	0.0012	0.0012
19					0.0025	0.0025
22						0.0035

Table S6

pos(Imax) (xy)						
LDs	10	13	16	19	22	25
7	0.0003	0.0006	0.0012	0.0025	0.0006	0.0006
10		0.0003	0.0007	0.0016	0.0034	0.0152
13			0.0012	0.0025	0.0006	0.0006
16				0.0043	0.0012	0.0012
19					0.0025	0.0025
22						**0.3648**

Table S7

end(ED) th.=0.1						
LDs	10	13	16	19	22	25
7	0.0003	0.0006	0.0012	0.0025	0.0006	0.0006
10		0.0003	0.0007	0.0016	0.0003	0.0003
13			0.0012	0.0025	0.0175	0.0006
16				0.0043	0.0093	**0.2949**
19					0.0025	0.0025
22						**0.2086**

Table S8

size(ED) th.=0.1						
LDs	10	13	16	19	22	25
7	0.0003	0.0006	0.0012	0.0025	0.0006	0.0006
10		0.0003	0.0007	0.0016	0.0003	0.0003
13			0.0012	0.0025	0.0006	0.0006
16				0.0087	**0.1014**	**0.7308**
19					0.0025	0.0025
22						**0.1282**

Table S5-S8. Wilcoxon rank sum test tables for the median values of the beginning (ini), position of the maximum (Imax), end and size of the expression domain (ED). These data were obtained from the intensity profiles extracted from the xy sum-of-slice projection for 10%threshold (th.=0.1) (significance level, α=0.05). Red values identifying non-significant differences between the medians, i.e. those cases in which the null hypothesis cannot be rejected.

Development • Supplementary information

Table S9

I_t th.=0.1						
LDs	10	13	16	19	22	25
7	0.3357	0.0023	0.0012	0.0051	0.2086	0.2593
10		0.0003	0.0007	0.0016	0.6943	0.0401
13			0.9452	0.4318	0.0006	0.0111
16				0.1775	0.0012	0.0023
19					0.0051	0.0480
22						0.0530

Table S10

Conc. th.=0.1						
LDs	10	13	16	19	22	25
7	0.0289	0.0728	0.8357	0.0480	0.0012	0.0175
10		0.0012	0.0080	0.8329	0.0140	0.3357
13			0.0734	0.0025	0.0006	0.0006
16				0.0173	0.0012	0.0047
19					0.0303	0.6389
22						0.0973

Table S9-S10. Wilcoxon rank sum test tables for the median values of the total intensity (I_t) and signal concentration (Conc.). These data were obtained from the expression domain measurement from the intensity profiles extracted from the xy sum-of-slice projection for 10% threshold (th.=0.1) (significance level, α=0.05). Red values identifying non-significant differences between the medians, i.e. those cases in which the null hypothesis cannot be rejected.

Development • Supplementary information
