# Supplementary material for: Two modes of gene regulation by TFL1 mediate its dual function in flowering time and shoot determinacy of Arabidopsis
Source: Development. 2023 Dec 7;150(23):dev202089. doi: 10.1242/dev.202089 (PMC10730086; doi:10.1242/dev.202089)
Supplement: Supplementary file 1 [file develop-150-202089_review_history.pdf]

Two modes of gene regulation by TFL1 mediate its dual function in flowering time and shoot determinacy of Arabidopsis

Martina Cerise, Vitor da Silveira Falavigna, Gabriel Rodriguez Maroto, Antoine Signol, Edouard Severing, He Gao, Annabel van Driel, Coral Vincent, Sandra Wilkens, Francesca Romana Iacobini, Pau Formosa-Jordan, Alice Pajoro and George Coupland
DOI: 10.1242/dev.202089

Editor: Dominique Bergmann

Review timeline

Original submission:	16 June 2023
Editorial decision:	17 July 2023
First revision received:	2 November 2023
Accepted:	4 November 2023

Original submission

First decision letter

MS ID#: DEVELOP/2023/202089

MS TITLE: Two modes of gene regulation by TFL1 mediate its dual function in flowering time and shoot determinacy of Arabidopsis

AUTHORS: Martina Cerise, Vitor da Silveira Falavigna, Gabriel Rodriguez Maroto, Antoine Signol, Edouard Severing, He Gao, Annabel van Driel, Coral Vincent, Sandra Wilkens, Francesca Romana Iacobini, Pau Formosa-Jordan, Alice Pajoro, and George Coupland

I have now received all the referees reports on the above manuscript, and have reached a decision. The referees' comments are appended below, or you can access them online: please go to BenchPress and click on the 'Manuscripts with Decisions' queue in the Author Area.

The overall evaluation is positive and we would like to publish a revised manuscript in Development, provided that the referees' comments can be satisfactorily addressed. Please attend to all of the reviewers' comments in your revised manuscript and detail them in your point-by-point response. If you do not agree with any of their criticisms or suggestions explain clearly why this is so. If it would be helpful, you are welcome to contact us to discuss your revision in greater detail. Please send us a point-by-point response indicating your plans for addressing the referees' comments, and we will look over this and provide further guidance.

Reviewer 1

Advance summary and potential significance to field

The ms. by Cerise et al is a remarkable piece of work where the authors perform a comprehensive and rigorous study of the dynamics of TFL1 in the shoot apical meristem of Arabidopsis at the floral transition that will help many researchers working in the field of flowering time and inflorescence development regulation.

The study presented here is, however, largely descriptive. The conclusions of the work are somehow limited in terms of major advances in our knowledge of these processes. In any case, the

© 2023. Published by The Company of Biologists under the terms of the Creative Commons Attribution License (https://creativecommons.org/licenses/by/4.0/).

quality of the data and the tools generated are more than sufficient to fuel and support subsequent studies and they are also likely to help to reinterpret some of our views of the function of TFL1 and FT. In summary, a wealth of data is provided that, in my opinion, should be very useful to the research in the field.

Comments for the author

I really cannot see major issues with the results or the discussion of the data presented here, and I do not have significant concerns that should be addressed. However, I think that there are a few points that could be better explained to improve a bit the ms.

1. L29. What is the (molecular) evidence of TFL1 being a corepressor? I believe that it is still at debate whether it is simply a competitor of FT or really blocks transcription.
2. L161. I am intrigued about the expression of TFL1 in the axillary meristems. I guess that at least some are at the axils of cauline leaves, that are supposed to have I1 identity too. Do these meristems dome like the SAM?, Does TFL1 have a similar dynamic behaviour (deep- diffuse- tip)? How is the overlap with FT in those?
3. The cluster 2 and cluster 3 DEGs discussed in the paper correlate quite well with the propose dual function of TFLL1 in controlling flowering time and inflorescence meristem identity. However, I think it would have been easier to separate these roles in a parallel study in SD, where TFL1 function in meristem determinacy does not overlap so much in time (tfl mutants make more flowers before termination). Have these experiments been attempted? Can some of the relevant DEGs in cluster 2 and 3 at least be tested in these conditions to really support their different behaviour?
4. L286. The complementary patterns of SOC1 and AP1 in the SAM of tfl1 are discussed, in terms of AP1 repressing SOC1. However, FUL, described before as repressed by AP1, is still present in these meristems.
What could be the reason for this behaviour?
5. L327. SEP4 emerges as an interesting participant in both roles of TFL1, different from other SEP genes. If TFL1 is just a competitor of FT for FD, how is SEP4 regulated in ft mutants? are ther eany data that could be provided here?
6. L360. I do not understand well the experiments testing the expression on leaves of FT and other genes in the tfl1 mutant. If TFL1 is expressed at very low levels in leaves, would it be more informative to test in the meristem? I think this should be tested as it is not a cumbersome experiment and, at leats to me, it makes more sense 7. L438. I guess that FT-VENUS is not an option, but to really support the “competition” hypothesis between FT and TFL1 for FT, it would be necessary to test the FT acumulation pattern at the SAM when TFL1 becomes diffuse. Otherwise, is quite speculative.

<u>Reviewer 2</u>

Advance summary and potential significance to field

This work presents new data on TFL1, an important regulator of inflorescence development that prevents early flowering and conversion of the shoot apical meristem into a terminal flower. This regulator exist and play key roles in a wide variety of angiosperms and precisely understanding how it performs these different functions is an important developmental question.

This work represents a clean, well-designed and concise study combining temporal series using morphological characterisations, fluorescent markers lines to analyse protein (co-expression) patterns and gene expression (RNA-seq). Finally, characterisations of multiple mutant complements these analyses by analysing the roles of individuals actors including some identified as playing a possible role during this process.

The major finding is that TFL1 has an interesting expression pattern before and after meristem doming and that it acts on two clusters of genes that are each associated with early flowering or early termination. The identification of SEP4 (and not the closely related 3 other SEPs) as a major actors from cluster 3 is a very interesting result, well supported buy the data.

© 2023. Published by The Company of Biologists under the terms of the Creative Commons Attribution License (https://creativecommons.org/licenses/by/4.0/).

Overall, even if this manuscript is focused on a precise question, I think it is very valuable for the novelty of the findings (2 domains/2 target gene clusters), the solidity (and the beauty) of the in vivo analysis (fluorescent lines and in situ hybridisation) and the high quality of the genetic data.

Comments for the author

Comments

- the time resolved transcriptomic analyses has to be explained more clearly. I have struggled very hard to understand the § starting at line 175. I am aware it is always a challenge to explain comparison when dealing with time series and mutant vs wt but the way it is presented here is way to tough on the reader. The term DEG needs to be defined very well (temporal series, which reference or mutant at which time point) or used with an indication of the nature of the comparison. I suggest adding to the Venn diagram a figure with numbers explaining the different comparisons that are made and then number of genes they yield.
- the differences between SEP3 and SEP4 are discussed in terms of protein properties. Do we know different in their capacity to form tetramers with other MADS TF that could explain these differences ? Couldn’t the differences be rather due to expression and regulation of these genes and in this case, could differences in their regulatory sequences be responsible for the difference role.
- since TFL1 is important in many species, it would be interesting to add some evolutionary aspects in the discussion. I indicated here an important review I was surprised not to see cited. https://doi.org/10.1016/j.tplants.2019.02.004
- A few words about other genes present in the cluster could also be interesting
- I think these 2 last points could be discussed to enrich the discussion that remains quite close from the experiments

minor points
- 2 typos at line 56
- Line 97 is bizarre as it presents a side question as if it was the main one in the paper
- Continuous long day = continuous light or LD ?
- Line 323: « genetic interactions between SEP genes and tfl1 mutation « sounds awkward. Interaction between genes or between mutations would be more correct.
- Why not add SEP3 to the very nice summary of expression patterns present in the supp. materials? Along this line, a little schematic summary would be welcome in the last figure

---

First revision

<u>Author response to reviewers' comments</u>

Response to the editor and reviewers

We have responded to each of the referees’ points below. The referees’ comments are shown verbatim, and our responses are in bold. The line numbers mentioned refer to the clean Word file. All changes are marked in the tracked Word file that we have also uploaded. We would like to take this opportunity to thank the editor and referees for their constructive comments, which have helped to improve the manuscript.

Editor
The overall evaluation is positive and we would like to publish a revised manuscript in Development, provided that the referees' comments can be satisfactorily addressed. Please attend to all of the reviewers' comments in your revised manuscript and detail them in your point-by-point response. If you do not agree with any of their criticisms or suggestions explain clearly why this is so. If it would be helpful, you are welcome to contact us to discuss your revision in

© 2023. Published by The Company of Biologists under the terms of the Creative Commons Attribution License (https://creativecommons.org/licenses/by/4.0/).

greater detail. Please send us a point-by- point response indicating your plans for addressing the referee's comments, and we will look over this and provide further guidance.

We were pleased to be invited to submit a revised version. We have uploaded a clean version of the revised manuscript and one in which our changes are indicated with track changes.
In addition, we would like to mention that all of the raw data generated for this manuscript have now been uploaded to Dryad under the temporary link:
https://datadryad.org/stash/share/7IwS_Ao3NyHnXFgdwe8EoiUXJLEFp7rXXZE06gS tyLg
This link is available for the reviewers and editor. The final link that should be used if the manuscript is accepted for publication is mentioned in the main text but has not yet been activated.

Reviewer 1
Advance Summary and Potential Significance to Field:
The ms. by Cerise et al is a remarkable piece of work where the authors perform a comprehensive and rigorous study of the dynamics of TFL1 in the shoot apical meristem of Arabidopsis at the floral transition that will help many researchers working in the field of flowering time and inflorescence development regulation.

The study presented here is largely descriptive and the conclusions of the work provide moderate advances in our knowledge of these processes. But the quality of the data and the tools generated are more than sufficient to fuel and support subsequent studies and they are also likely to help to reinterpret some of our views of the function of TFL1 and FT. In summary, a wealth of data is provided that, in my opinion, should be very useful to the research in the field.
We thank the reviewer for the positive comments on our work.

Comments for the Author:
I really cannot see major issues with the results or the discussion of the data presented here, and I do not have significant concerns that should be addressed. However, I think that there are a few points that could be better explained to improve a bit the ms.
We thank the reviewer for the positive comments on the data and discussion, and appreciate the suggestions for improvements to the manuscript. We have responded to each of the comments below.

1. L29. What is the (molecular) evidence of TFL1 being a corepressor? I believe that it is still at debate whether it is simply a competitor of FT or really blocks transcription.
Yes, the reviewer makes a good point that TFL1 reduces the transcript levels of FD targets, but this could occur by blocking access of FT rather than actively repressing transcription. Therefore, calling it a "transcriptional repressor" may be an overinterpretation. We have edited this to read "a negative regulator of transcription" (L29), which we think is general enough to include the possibilities that it blocks access of FT or acts as a transcriptional repressor.

2. L161. I am intrigued about the expression of TFL1 in the axillary meristems. I guess that at least some are at the axils of cauline leaves, that are supposed to have I1 identity too. Do these meristems dome like the SAM?, Does TFL1 have a similar dynamic behaviour (deep- diffuse- tip)? How is the overlap with FT in those?
Yes, TFL1 is expressed strongly in axillary meristems in the axils of cauline leaves, and is assumed to contribute to repressing floral meristem identity and therefore maintaining I1 identity in these meristems, because in *tfl1* mutants they are converted to flowers. To answer the points raised by the reviewer, it would be necessary to follow the morphology of the meristem and TFL1 expression as the axillary meristem grows out to form a bud and then a branch. The present analysis focused on the SAM and the nodes harboring fully developed axillary meristems and buds were removed during harvesting. Therefore, addressing the points raised by the reviewer requires a future project that would involve specifically harvesting and analyzing axillary meristems at appropriate stages. Our expectation is that as the axillary bud is activated and grows out, TFL1 expression will show a similar dynamic pattern as that we describe in the SAM, but this has not yet been tested. The relative patterns of accumulation of FT and TFL1 have also not been described in the axillary meristem or the SAM, but will be an interesting future direction.

© 2023. Published by The Company of Biologists under the terms of the Creative Commons Attribution License (https://creativecommons.org/licenses/by/4.0/).

3. The cluster 2 and cluster 3 DEGs discussed in the paper correlate quite well with the propose dual function of TFLL1 in controlling flowering time and inflorescence meristem identity. However, I think it would have been easier to separate these roles in a parallel study in SD, where TFL1 function in meristem determinacy does not overlap so much in time (tfl mutants make more flowers before termination). Have these experiments been attempted? Can some of the relevant DEGs in cluster 2 and 3 at least be tested in these conditions to really support their different behaviour?

We studied the role of TFL1 under long days because FT is expressed under these conditions, and therefore the antagonism between FT and TFL1 is much more evident under these conditions, and the effect of the *tfl1* mutation is much stronger. We did study the pattern of expression of *gTFL1-VENUS* under continuous short days (Supplementary Figure 2.1C), and reported the same dynamic pattern of expression detected under LDs, suggesting the two roles of TFL1 are separable under SDs. However, the reviewer is correct that we did not compare the patterns of expression of cluster 2 and 3 genes under these conditions and nor did we analyse *tfl1-18* meristem shape under this condition. We have now extracted an RNA time course under SDs and in parallel harvested SAMs to verify the morphology of the *tfl1-18* mutant compared to wild type in this condition. These data are shown in Supplementary Figure 3.5, and described in the Results from L228 to L240). We observed that inflorescence meristems of both *tfl1-18* and Col-0 were first formed at 6 weeks after germination under SD, consistent with tfl1-18 having a minor effect on flowering time under SD. Accordingly none of the genes of Cluster 2 tested (*FUL*, *SEP4*, *SPL3* and *SPL8*) changed in expression between Col-0 and *tfl1-18* under SDs. Moreover, we showed that in *tfl1-18* under SD a terminal flower is formed at 10 weeks and that at 8 weeks under SD some of the tested Cluster 3 genes were increased in mRNA level in *tfl1-18* compared to the wild type (*AP1*, *AP3*, *SEP1*, *SEP2*). These data suggest that under SDs the genes of Cluster 2 have a mild or no effect on the *tfl1-18* flowering-time phenotype, which is indeed weaker in SD than in LD, probably because FT is not expressed under SD. Nevertheless, several Cluster 3 genes are increased in expression soon before terminal flower formation under these conditions. A more detailed analysis of *TFL1* function and the activities of these two gene clusters under SDs would require more detailed analyses of quantifying traits such as shoot and meristem morphology through a time course under SDs, and analysing the genome-wide transcriptome.

4. L286. The complementary patterns of SOC1 and AP1 in the SAM of tfl1 are discussed, in terms of AP1 repressing SOC1. However, FUL, described before as repressed by AP1, is still present in these meristems. What could be the reason for this behaviour?

The reviewer is right that *gFUL-VENUS* does not appear to be repressed by AP1 in the SAM of *tfl1-18* mutants. At this point, we cannot explain the difference in regulation of SOC1 and FUL. One possibility is that the repression of *FUL* by AP1 requires other transcription factors that are not misregulated in the SAM of *tfl1- 18* and are not required for *SOC1* repression by AP1. We have now drawn attention to this point by adding the sentence "However, *gFUL:VENUS* expression appears not to be repressed by AP1 in the inflorescence SAM (Fig. 4A)" (line 306-307 in tracked version). We have also made it clear that AP1 does not repress all genes involved in floral transition in the SAM by inserting "some" in the sentence "*AP1*, appears to alter the spatial and temporal expression patterns of some genes involved in the promotion of floral transition or inflorescence development that are normally expressed in the SAM of Col-0." (line **309 in tracked version).**

5. L327. SEP4 emerges as an interesting participant in both roles of TFL1, different from other SEP genes. If TFL1 is just a competitor of FT for FD, how is SEP4 regulated in ft mutants? are there any data that could be provided here?

As the reviewer mentioned, in the manuscript we provided transcriptome and genetic evidence that *SEP4* has a different role than other *SEPALLATA* genes in *tfl1-18*. Previous analysis showed that *SEP1*, *SEP2* and *SEP3* are reduced in expression in *ft* mutants compared to the wild type (Schmid et al. 2003; https://doi.org/10.1242/dev.00842). Recently, RNA-seq of *ft tsf* enriched meristems was performed in our group and preliminary analysis showed that *SEP4* is downregulated in *ft tsf* double mutants. Providing all of these unpublished RNAseq data would be a distraction for this manuscript, but we have provided a plot of *SEP4* mRNA in

© 2023. Published by The Company of Biologists under the terms of the Creative Commons Attribution License (https://creativecommons.org/licenses/by/4.0/).

Col-0 and *ft tsf* as part of this response (data from Pedro de los Reyes).

NOTE: We have removed unpublished data that had been provided for the referees in confidence.

6. L360. I do not understand well the experiments testing the expression on leaves of FT and other genes in the tfl1 mutant. If TFL1 is expressed at very low levels in leaves, would it be more informative to test in the meristem? I think this should be tested as it is not a cumbersome experiment and, at leats to me, it makes more sense

The reviewer suggested to check the expression of *FT* and the other genes related to FT induction in the meristem of *tfl1-18*. Indeed, this is an interesting suggestion because the lack of *TFL1* in this tissue could cause the misexpression in the SAM of flowering genes normally expressed in the leaves. We included these data in Suppl. Fig. 6.1 showing the mRNA levels of *FT*, *TFL1*, *FD*, *CO*, *GI and SOC1* extracted from the RNA-seq of apices of Col-0 and *tfl1-18* plants grown under LDs. As reported now in lines 377-387, we observed that *FT* expression is absent also in *tfl1-18* apices, but we did find that *FD* and *CO* mRNAs are slightly upregulated in *tfl1-18* at individual time points. The higher level of FD in apices could contribute to enhanced FT response, and we have added this point on line 385 - 387.

7. L438. I guess that FT-VENUS is not an option, but to really support the "competition" hypothesis between FT and TFL1 for FT, it would be necessary to test the FT acumulation pattern at the SAM when TFL1 becomes diffuse. Otherwise, is quite speculative.

Yes, we agree with the reviewer that this is the next step. However, it requires translational fusions to FT expressed from the complex *FT* transcriptional signals that properly complement the *ft* mutation, and we are working on that but it has been difficult to identify appropriate lines and will require further time. While we agree with the reviewer that the "competition" hypothesis is speculative at this stage, it is raised only as a possibility in the Discussion, and we would prefer to retain it. However, we have added the point made by the reviewer that this possibility should be tested by imaging *gFT* translational fusions in Col-0 and *ft* mutants in the Discussion (line 472-475).

Reviewer 2

Advance Summary and Potential Significance to Field:

This work presents new data on TFL1, an important regulator of inflorescence development that prevents early flowering and conversion of the shoot apical meristem into a terminal flower. This regulator exist and play key roles in a wide variety of angiosperms and precisely understanding how it performs these different functions is an important developmental question.

This work represents a clean, well-designed and concise study combining temporal series using morphological characterisations, fluorescent markers lines to analyse protein (co-expression) patterns and gene expression (RNA-seq). Finally, characterisations of multiple mutant complements these analyses by analysing the roles of individuals actors including some identified as playing a possible role during this process.

The major finding is that TFL1 has an interesting expression pattern before and after meristem doming and that it acts on two clusters of genes that are each associated with early flowering or early termination. The identification of SEP4 (and not the closely related 3 other SEPs) as a major actors from cluster 3 is a very interesting result, well supported buy the data.

Overall, even if this manuscript is focused on a precise question, I think it is very valuable for the novelty of the findings (2 domains/2 target gene clusters), the solidity (and the beauty) of the in vivo analysis (fluorescent lines and in situ hybridisation) and the high quality of the genetic data.

We thank the reviewer for the positive comments on our work.

Comments for the Author:

- the time resolved transcriptomic analyses has to be explained more clearly. I have struggled very hard to understand the § starting at line 175. I am aware it is always a challenge to explain comparison when dealing with time series and mutant vs wt but the way it is presented here is way to tough on the reader. The term DEG needs to be defined very well (temporal series, which

© 2023. Published by The Company of Biologists under the terms of the Creative Commons Attribution License (https://creativecommons.org/licenses/by/4.0/).

reference or mutant at which time point) or used with an indication of the nature of the comparison. I suggest adding to the Venn diagram a figure with numbers explaining the different comparisons that are made and then number of genes they yield.
Yes, we understand that the comparisons of genotype and time point might have been difficult to grasp from the original descriptions. We have thoroughly edited the text as suggested by the reviewer. In the section between lines 176 and 194, we have reduced the length and complexity of the sentences and tried to make only one point in each sentence. We hope that these changes make the RNA-seq analyses performed clearer. We have also followed the suggestion of the reviewer and created a new Suppl. Fig. 3 scheme to visually illustrate the comparisons made to reach the final list of genes.

- the differences between SEP3 and SEP4 are discussed in terms of protein properties. Do we know different in their capacity to form tetramers with other MADS TF that could explain these differences? Couldn't the differences be rather due to expression and regulation of these genes and in this case, could differences in their regulatory sequences be responsible for the difference role.

The reviewer is right that we mention in the Discussion that previous work demonstrated that both SEP3 and SEP4 could form dimers with FUL and SOC1. Differences between SEP4 and SEP3 in tetramer formation are also possible but have not as far as we know been tested. The different transcript patterns we describe do suggest differences in their regulation, but in *tfl1-18* mutants both *SEP3* and *SEP4* are expressed in the SAM, but only *SEP4* has an effect on floral transition. We therefore discuss that this could be due to differences in their biochemical function. We have edited the text of the Discussion (line 514- 530) to make it clear that in this case there would be differences both in their transcriptional regulation and the biochemical functions of their proteins.

- since TFL1 is important in many species, it would be interesting to add some evolutionary aspects in the discussion. I indicated here an important review I was surprised not to see cited. https://doi.org/10.1016/j.tplants.2019.02.004

We thank the reviewer for the suggestion. There are many important analyses of the functions of TFL1 orthologues in different species, and we deleted this previously because of space constraints. We have now included a paragraph at the beginning of the Discussion that mentions some examples of TFL1 orthologues in other species and the review indicated by the reviewer (Line 395-403). We have framed the paragraph in terms of the dual function of TFL1 in controlling flowering time and inflorescence development of Arabidopsis, and that this is not always conserved or is conferred by different paralogues in other species.

- A few words about other genes present in the cluster could also be interesting

Yes, we agree that only a few genes for each Cluster were reported in the Results. We have now added in Suppl. Fig. 3.4 more genes from Cluster 2 and 3. We also included some of them in the new analysis we performed under SD conditions. Suppl Fig.3.4 showing additional genes in the Clusters is mentioned in the Results on line 217 and line 228.

- I think these 2 last points could be discussed to enrich the discussion that remains quite close from the experiments

As mentioned above, we have incorporated a paragraph on TFL1 in other species in the Discussion, and briefly mention other genes. However, we have not added a long discussion on other genes as this could be a distraction from the main points.

minor points
- 2 typos at line 56

Thank you, we found and corrected these.

- Line 97 is bizarre as it presents a side question as if it was the main one in the paper

We deleted that sentence.

- Continuous long day = continuous light or LD ?

We deleted continuous. It is a term we use to indicate exposure to long days continuously through the life cycle of the plant, but was unnecessary here.

© 2023. Published by The Company of Biologists under the terms of the Creative Commons Attribution License (https://creativecommons.org/licenses/by/4.0/).

- Line 323: « genetic interactions between SEP genes and tfl1 mutation « sounds awkward. Interaction between genes or between mutations would be more correct.
Thank you, we edited this as suggested.

- Why not add SEP3 to the very nice summary of expression patterns present in the supp. materials? Along this line, a little schematic summary would be welcome in the last figure

We thank the reviewer for these suggestions. We added in Suppl. Fig. 4.2 both *SEP3* and *SEP4* expression, and mentioned the Figure on line 286-287 (tracked version). We found it difficult to summarise the results in a small schematic in the final figure, and prefer to rely on the summary in the text for example the Abstract and Discussion and the schematic of the misexpression patterns in Suppl. Fig. 4.2.

Second decision letter

MS ID#: DEVELOP/2023/202089

MS TITLE: Two modes of gene regulation by TFL1 mediate its dual function in flowering time and shoot determinacy of Arabidopsis

AUTHORS: Martina Cerise, Vitor da Silveira Falavigna, Gabriel Rodriguez Maroto, Antoine Signol, Edouard Severing, He Gao, Annabel van Driel, Coral Vincent, Sandra Wilkens, Francesca Romana Iacobini, Pau Formosa-Jordan, Alice Pajoro, and George Coupland
ARTICLE TYPE: Research Article

I am happy to tell you that your manuscript has been accepted for publication in Development, pending our standard ethics checks.

© 2023. Published by The Company of Biologists under the terms of the Creative Commons Attribution License (https://creativecommons.org/licenses/by/4.0/).
